# Spatial and Temporal Characteristics of Pastoral Mobility in the Far North Region, Cameroon: Data Analysis and Modeling

**DOI:** 10.1371/journal.pone.0131697

**Published:** 2015-07-07

**Authors:** Ningchuan Xiao, Shanshan Cai, Mark Moritz, Rebecca Garabed, Laura W. Pomeroy

**Affiliations:** 1 Department of Geography, The Ohio State University, Columbus, OH, United States of America; 2 Department of Geography, Nipissing University, North Bay, Ontario, Canada; 3 Department of Anthropology, The Ohio State University, Columbus, OH, United States of America; 4 Netherlands Institute for Advanced Study (NIAS), Wassenaar, the Netherlands; 5 Department of Veterinary Preventive Medicine, The Ohio State University, Columbus, OH, United States of America; Southwest University, CHINA

## Abstract

Modeling the movements of humans and animals is critical to understanding the transmission of infectious diseases in complex social and ecological systems. In this paper, we focus on the movements of pastoralists in the Far North Region of Cameroon, who follow an annual transhumance by moving between rainy and dry season pastures. Describing, summarizing, and modeling the transhumance movements in the region are important steps for understanding the role these movements may play in the transmission of infectious diseases affecting humans and animals. We collected data on this transhumance system for four years using a combination of surveys and GPS mapping. An analysis on the spatial and temporal characteristics of pastoral mobility suggests four transhumance modes, each with its own properties. Modes M1 and M2 represent the type of transhumance movements where pastoralists settle in a campsite for a relatively long period of time (≥20 days) and then move around the area without specific directions within a seasonal grazing area. Modes M3 and M4 on the other hand are the situations when pastoralists stay in a campsite for a relatively short period of time (<20 days) when moving between seasonal grazing areas. These four modes are used to develop a spatial-temporal mobility (STM) model that can be used to estimate the probability of a mobile pastoralist residing at a location at any time. We compare the STM model with two reference models and the experiments suggest that the STM model can effectively capture and predict the space-time dynamics of pastoral mobility in our study area.

## Introduction

Humans and animals constantly move from one place to another. Modeling such movements is critical for understanding the transmission of infectious diseases in complex social-ecological systems [1–5]. In this research we focus on a special human mobility system called transhumance, a common practice where pastoralists move their livestock seasonally across different grazing lands [6]. Researchers distinguish between different forms of transhumance, including vertical transhumance from winter pastures in the valleys to summer pastures in the mountains [7] and horizontal transhumance, for example, from rainy season pastures in the northern Sahel to dry season pastures in the southern Sahel [8]. Transhumance patterns vary in terms of the distance covered, the number of movements, the duration of stays in each location, and the direction of movement when the season changes. While these movements are beneficial in terms of optimizing the use of land and other natural resources [9, 10], they also may play a role in disease transmission [11–17] and have other significant regional or local social/economic impacts [18, 19]. Understanding the spatial and temporal patterns of these transhumant systems will help us understand the role of such movements in the transmission of infectious diseases. For example, the specific movement patterns of animals in conjunction with demographic patterns (e.g., birthing seasons) and environmental conditions (e.g., wet versus dry and cold versus hot) could result in significant changes in whether transmission of infectious diseases is prevented or facilitated by movements [20, 21].

There are different spatial and temporal levels to analyze pastoral mobility, ranging from daily movements to pasture and water [22, 23], to seasonal transhumance movements between rainy and dry season grazing areas [8, 24], and migration across country borders at the scale of decades [6, 25, 26]. Empirical research has shown that pastoral mobility is a highly efficient and sustainable strategy to cope with spatial and temporal variation in grazing resources that is typical in arid and semi-arid ecosystems [27–30]. While researchers have been studying pastoral mobility for long [6, 7], the use of GPS and mapping technology has facilitated the study of pastoral mobility enormously [31, 32] through the use of hand-held devices and/or tracking devices [33]. However, because of the challenges involved in following multiple herds distributed over large rangelands, most studies track only relatively few individual households, ranging from one [34] to twenty-four [31]. These small samples may not be representative of the movements of the larger population of mobile pastoralists in a region. Descriptions of transhumance patterns at the population level are often too general and are simply indicated with broad arrows on a map [35]. What also remains unclear is whether and how transhumance patterns affect the transmission of infectious diseases, but to examine that we first need to describe and model transhumance patterns of the pastoral population at a regional scale.

In this paper, we study human and animal mobility focusing on the transhumance of mobile pastoralists in the Far North Region of Cameroon. One of the main reasons we model the movements of these pastoralists is to understand the potential role of pastoral mobility in the risks of spreading infectious diseases, in particular foot-and-mouth disease [36, 37], in the region. We address two key questions: (1) what are the fundamental spatial and temporal characteristics of the mobile pastoralists’ transhumance movements in the region, and (2) how to develop a statistical model that can be used to describe these movements? Our goal is to develop a model that can be used to predict transhumance movements. In other words, what is the probability that a pastoralist will appear at a specific location at a specific time? Answers to these questions will effectively help us link pastoralist movements to the rest of the regional population and their herds in the region and thus help model disease transmission.

Many methods developed in the space-time geography and ecology literature are related to this research. The traditional space-time geography [38] focused on the analysis of the potential areas (called space-time prisms) an individual can reach between the origin and destination locations given a time budget and speed [39–41]. In reality, individuals do not visit every location in the potential area equally. Instead, some locations have a higher probability to be visited than others. In recognizing such heterogeneity, researchers utilized kernel density estimation methods [42] to generate surfaces of probability of an individual visiting a location in an area [43, 44]. Though kernel density models provide more details about the space-time activities of individuals, these models do not account for directional movements that are common in many forms of human mobility. In the ecology literature, researchers have used Brownian bridge motion model to describe animal movements so that we can estimate the location of animals at a time in the area around a migration route [45]. All these methods provide valuable context to this research. However, as will be demonstrated below by an analysis of our data, pastoralist transhumance patterns exhibit more heterogeneous modes that may not be captured using a single model and it is necessary to develop a new approach to modeling space-time behavior of the pastoralists. In the remainder of the paper we first discuss our study area and the data collecting process. Then we discuss the methodological details of analyzing the data and building a spatial-temporal mobility model to capture the dynamics of pastoral mobility in the region. Two reference models are developed and are compared with the spatial-temporal model. We discuss how the three models are fitted using the data and demonstrate the use of these models in simulating transhumance in the region. The performance of the three models is tested using a subset of our data. We conclude the paper with a discussion on a broader context of the analysis and modeling of mobility data.

## Study Area and Data

**Fig 1:**
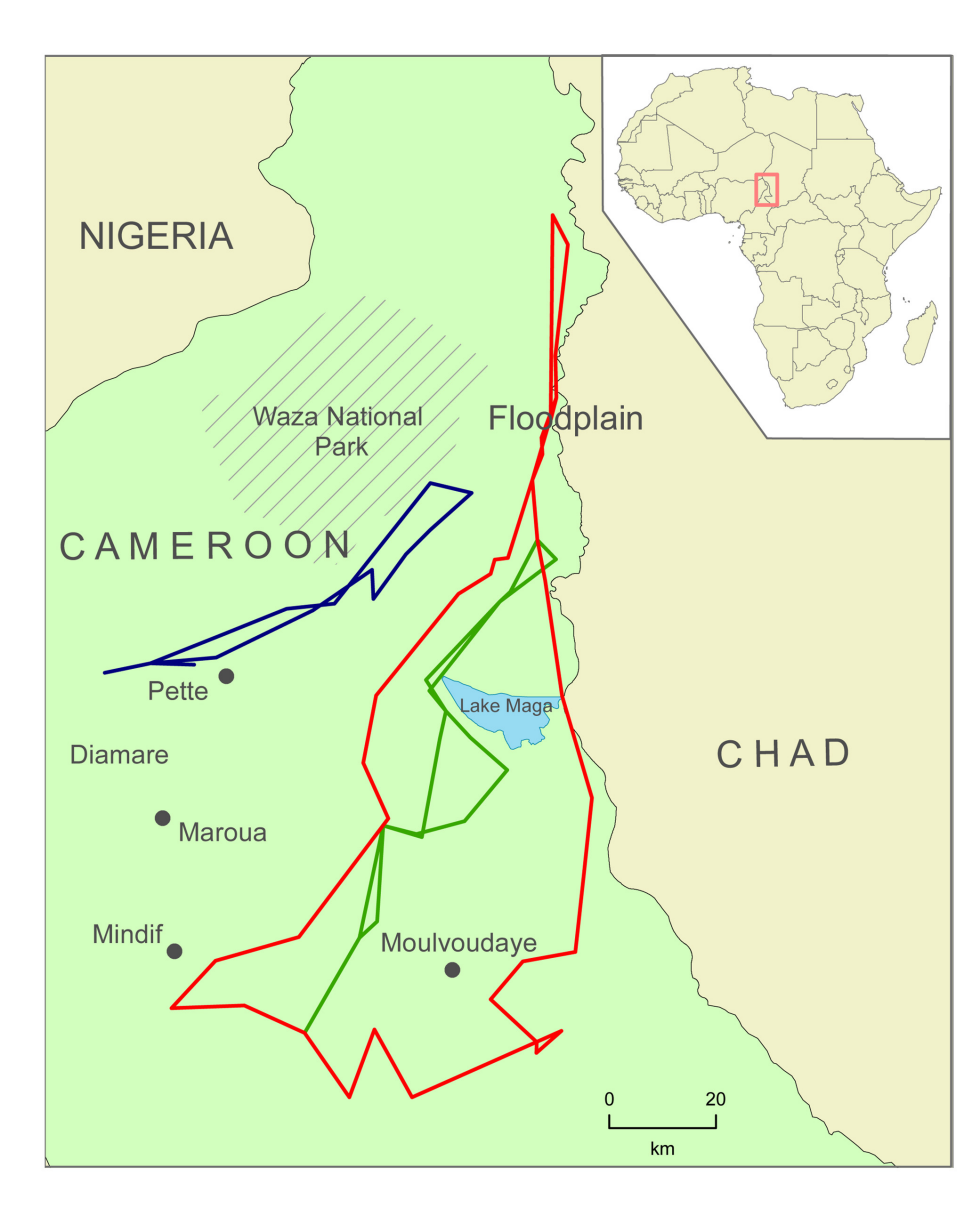
The study area and three sample transhumance routes. The base map is prepared using the public domain data of 1:50 Million world countries from Natural Earth.

The Logone Floodplain is one of the most important dry season grazing areas in the Chad Basin, supporting more than 200,000 cattle [46]. The floodplain is flooded by the Logone River and its branches in the rainy season and mobile pastoralists from the Far North Region of Cameroon and nearby countries (Chad, Nigeria, and Niger) find nutritious regrowth and surface water in the floodplain after the water recedes elsewhere in the dry season that typically starts in November. Many pastoralists remain there until the start of the rainy season in June, while others move to the grazing lands south to Lake Maga. At the start of the rainy season, pastoralists return to the higher elevated dunes in Diamaré in Cameroon or their respective countries [47]. Fig 1 shows the overall environment of the region along with three sample transhumance routes used by Cameroonian pastoralists in the year of 2007–2008.

Seasonal change in rainfall is the major force that drives pastoralists to seek forage and water for their cattle. In their transhumance process, the pastoralists set up camps and stay at each campsite for a number of days. There are two main kinds of campsites: *sojourn* campsites where the pastoralists stay for an extended period of time, and *transit* sites where they may only stay for a few days. We conducted a transhumance survey to reconstruct the annual transhumance routes used by the mobile pastoralists in the study area. The survey was conducted in the rainy season on each camp and we asked the names and number of days of all the campsites the pastoralists had stayed in the previous year. While most pastoralists were able to recall with ease the campsites that they stayed, the days of the stay did not always add up to 365 days. By assuming the lack of accuracy was more likely to occur when the pastoralists reported their stays at sojourn sites, we adjusted the days for the locations where they stayed the longest. We also recorded the number of pastoral households, the number of herds, and estimated the size of the herds.

To map the locations of the camps of mobile pastoralists in our study area, our research assistants traveled to the reported campsites four times a year in February, May, August, and November. The campsites were straightforward to recognize in the field and we used GPS technology to get the coordinates. Some of the campsite locations were also confirmed and adjusted using Google Earth and Google Maps that have high resolution satellite images for some of the areas in our region. We used the data collected in the mapping of mobile pastoralists to create a list of all the campsites (sojourn and transit) in our study area. The list currently has more than 400 campsites. A campsite typically consists of multiple households that during the year may split and some households may join other camps. Some campsites may not be available for our survey at some years. As a result, the composition of these camps changes throughout the year and the total number of campsites in our data varies from year to year. Our finalized data set has 553 households and herds and 46,725 cattle (estimated based on the average number of cattle per herd per ethnic group [47]). At the time the work presented in this paper started, data from four years, 2007–2011, were available for analysis and modeling. There are 71, 62, 83, and 62 camps for the four years of 2007–08, 2008–09, 2009–10, and 2010–11, respectively. This data set has been used in multiple previous studies [10, 23, 48].

### Ethics Statement

The research conducted in Cameroon was permitted by the Ministry of Scientific Research and Innovation of Cameroon and administered by the Garoua Wildlife College and the Higher Institute of the Sahel at the University of Maroua. Access to pasture lands is public in the Far North of Cameroon and does not require specific permission. We did not record participant consent. We used a verbal consent script and have obtained a waiver to document consent from the Institutional Review Board at the Ohio State University. The main reason for not documenting consent is that most informants are illiterate and documentation of consent is threatening and alienating in the cultural context in which we conduct our research. The participation in the research is voluntary and there is no consequences of non-participation. The private data and personally identifiable information are recorded separately. The transhumance survey protocol and informed consent procedure were approved by the Ohio State University Institutional Review Boards under #2010B0004 and #2007B0301.

## Analyzing Transhumance Modes

We define a *transhumance mode* as a set of characteristics related to the camping, grazing, and migration activities that are shared by a group of pastoralists during a certain period of time in an area. The mobile pastoralists in our study area clearly exhibited different kinds of transhumance activity that can be identified as distinctive transhumance modes. We started by examining the seasonality in the region. While the dry and rainy seasons are the two salient phytogeographic conditions in the region, the pastoralists also respond to smaller environment changes such as changing pasture conditions to bush fires or the presence of herds with infectious diseases. We identified the following five distinctive time periods (or seasons) in the Far North Region of Cameroon [23, 47].

- The period of July to September is the typical *rainy season* of the region and the mobile pastoralists mostly locate their herds in sojourn campsites in the areas of Mindif-Moulvoudaye and Pette. The largest amount of rainfall comes in July and August.
- In the *end of the rainy season*, generally in September, pastoralists start transhumance toward the floodplain where sufficient resources will be present in the rest of the annual cycle and the pastoralists tend to set up transit camps on their transhumance routes in this period of time.
- The *cold dry season* generally lasts from November to January. In this season, most pastoralists move around in the floodplain area.
- In the *hot dry season* that typically lasts from February to May, most pastoralists are settled in their sojourn campsites in the floodplain, while some have left for the areas south to Lake Maga where they establish sojourn sites to stay until the beginning of the rainy season.
- In June, a transition season called seeto or the *beginning of the rainy season*, pastoralists start to migrate back to the rainy season grazing lands.

**Fig 2:**
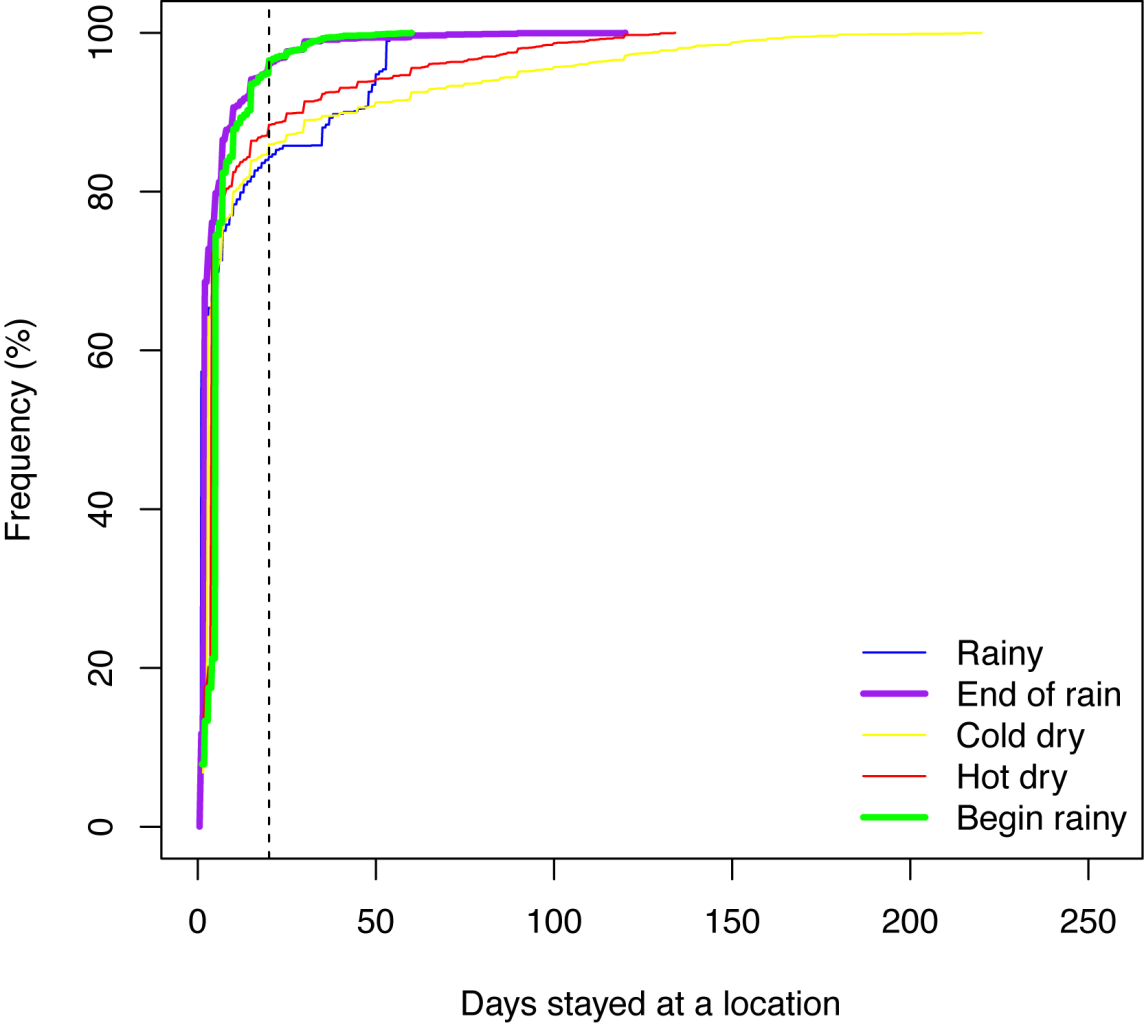
Cumulative distributions of the days of stay. The dashed vertical line indicates the stay of 20 days.

**Fig 3:**
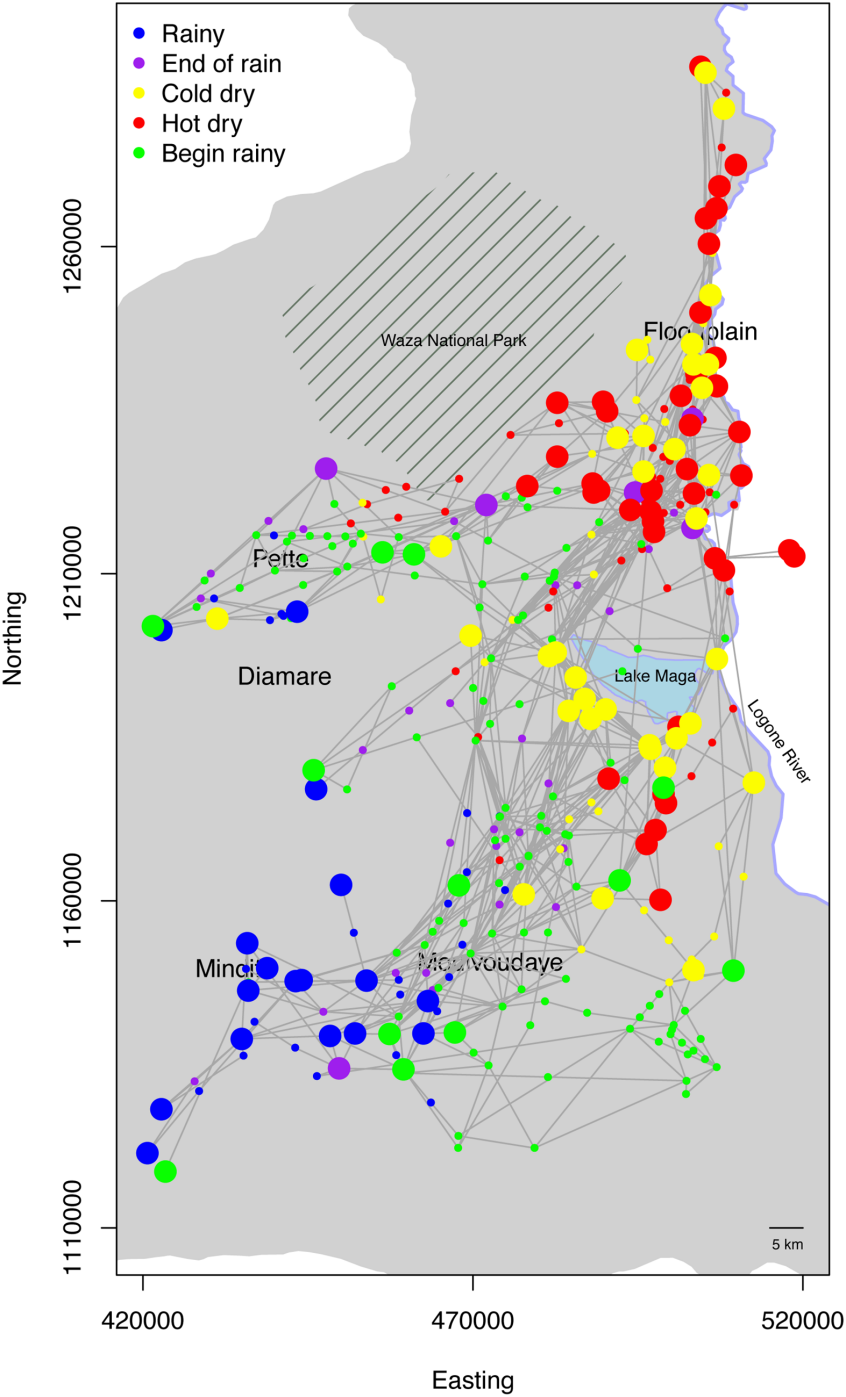
Transhumance routes and seasonality. The map shows all the pastoralists in year 2007–2008. Circles represent campsite locations and consecutive sites are linked using straight lines. Large circles represent sojourn campsites (≥ 20 days). Here the seasons are the time when the pastoralists start the camp. The shaded area shaded is part of the Far North Region.

Fig 2 shows the cumulative frequency of the duration the pastoralists stayed in each campsite in each season in the four years of our data. It is clear that in the end and beginning of the rainy season, the pastoralists tended to stay at each location in a relatively short amount of time as more than 95 percent of the stays are shorter than 20 days. On the other hand, in the rainy and dry (both cold and hot) seasons, many stays were longer as more than 15 percent of the stays were longer than 20 days. The 15 percent of the stays is significant, accounting for the majority of the days when these pastoralists stayed in their sojourn camps during these seasons. Fig 3 shows all the 71 transhumance routes in our data in year 2007–2008 with the locations of the campsites colored for the seasons, and the stays longer than 20 days are highlighted. We note the sojourn campsites were mainly located in the floodplain, the lands south to Lake Maga, and the rainy season lands in the area around Mindif and Diamaré.

**Fig 4:**
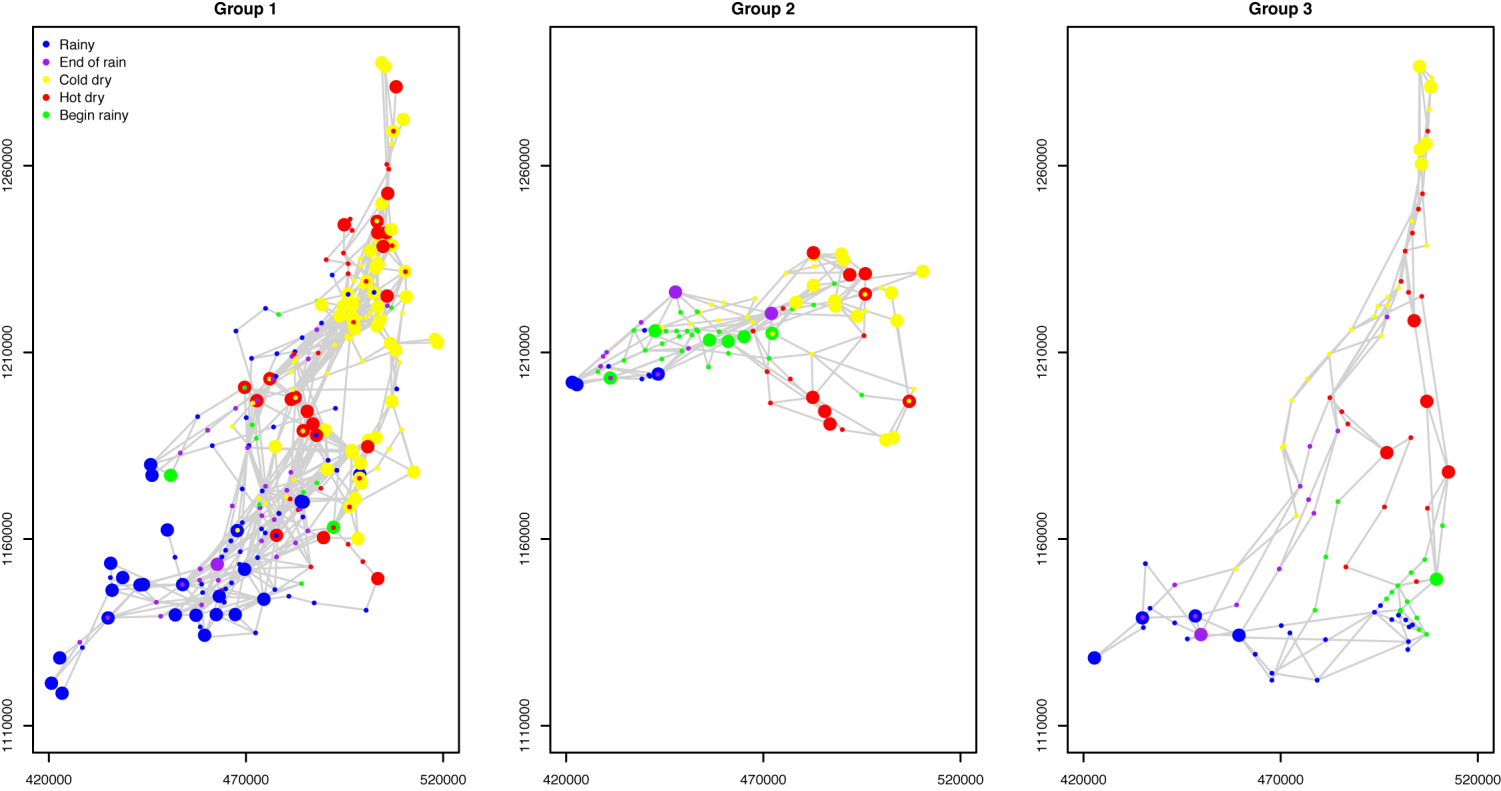
Three groups of transhumance routes.

The transhumance routes in Figs 1 and 3 show different overall shapes, paths, and rainy season locations. We used pastoralists’ toponyms and discussions of their transhumance routes to identify two important rainy season grazing areas: areas around Pette (referred to as Woylare by the pastoralists), and the Mindif-Moulvoudaye area (Fombina). Many pastoralists used relatively linear or straight transhumance routes. Most of the pastoralists who used the lands near Mindif-Moulvoudaye went back to their rainy season lands from the Floodplain through the paths west to Lake Maga (see the green route in Fig 1). Those who used areas near Pette as their rainy season lands had a similar linear shape of their routes (see the blue route in Fig 1). However, a small number of the pastoralists using the Mindif-Moulvoudaye area went back from the Floodplain through the paths east to Lake Maga (see the red route in Fig 1). Identifying these three groups of pastoralists is important to understand and to model pastoralist mobility because pastoralists in each group have similar spatial pattern (in routes and grazing areas) and temporal trends (in seasons). While there are other differences in these transhumance routes, these three groups are stable. Each pastoralist typically stayed in the same group over the years. In all the pastoralists surveyed in our data, only one changed the rainy season area (from Pette to Mindif). To quantitatively classify these patterns, we conducted a cluster analysis to distinguish the routes into three groups (see S1 Text for more methodological details). The first group included the pastoralists with their rainy season grazing lands around Mindif in the southwest part of the region and dry season lands in the floodplain (Fig 4, left). The second group included pastoralists who spent their rainy season in the Pette region (Fig 4, center). Both groups of pastoralists followed a relatively straight path between their seasonal locations. The third group, however, had a triangular shape.

**Fig 5:**
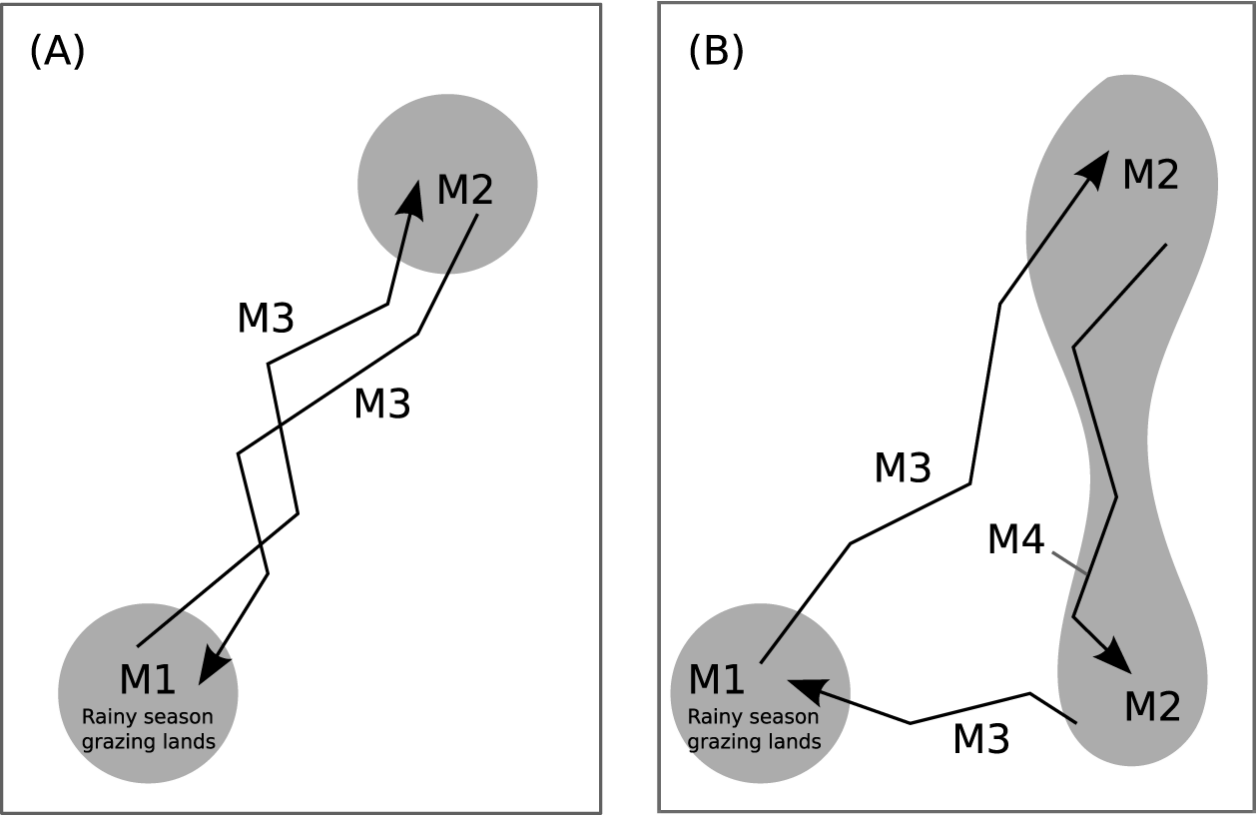
Transhumance modes for pastoralists in groups 1 and 2 (A) and group 3 (B).

The transhumance movements in the first two groups can be conceptualized in Fig 5A where they establish sojourn campsites in the south during the rainy season and we denote this type of transhumance mode as M1. In the end of the rainy season, the pastoralists start to move toward the floodplain and set up transit campsites en route to the floodplain. We denote their transhumance mode in this period of time as M3. In the floodplain, the pastoralists generally stay there over the cold and hot dry seasons, until the beginning of the rainy season. They utilize sojourn campsites during this period of time and we denote this type of transhumance as M2. We separate modes M1 and M2 because they occur in different parts of the region and in different seasons. Finally when the rain starts in June, pastoralists start to move back to their southern lands and we use M3 again to denote their transhumance patterns because the pastoralists use similar routes but in an opposite direction as they move in the end of the rainy season.

The third group of pastoralists have a more complicated transhumance pattern in space and time (Fig 5B). They still exhibit the M1 mode in the south. But their transhumance in the floodplain and the Lake Mega area during the cold and hot dry seasons have both sojourn and transit campsites, as shown in both large and small circles in the right map in Fig 4. We do not find a clear cut to separate the sojourn camps in the north and south and therefore use M2 to indicate the mode of using sojourn sites for long stays. In the mean time, some pastoralists in this group also set up transit camps as then move to the south and we add a new notation of M4 to denote this mode. They still use the M3 mode when they leave and come back to their rainy season grazing lands in the southwest part of the region.

Table 1 summarizes our notation system. Modes M1 and M2 are fundamentally different from modes M3 and M4. A pastoralist in M1 or M2 concerns relatively short movements in a relatively small area and may choose to stay at one location for a long period of time and then to move to a new location for the herd to graze. In modes M3 and M4, however, the pastoralist has a sense of moving toward the destination of a dry or rainy season area where grass is abundant, which forces the pastoralist to follow a clear, directional path.

**Table 1:** Transhumance modes.

Modes		Season	Campsite type	Months	Dates*
Group 1, 2	Group 3				
M1	M1	Rainy	Sojourn	July-August	1–17, 321–365
M3	M3	End of rains	Transit	September-October	18–78
M2	M2, M4	Cold dry	Sojourn, Transit (group 3)	November-January	79–170
M2	M2, M4	Hot dry	Sojourn, Transit (group 3)	February-May	171–290
M3	M3	Begin rainy season	Transit	June	291–320

*Dates are continuously numbered with day 1 starting on August 16.

## Modeling mobility

While the data analysis described in the previous section provides useful insight about pastoral mobility, to effectively link patterns in the data to other factors in a complex social and ecological system, a model is necessary and critical [49, 50]. We assume each pastoralist belongs to one of the three groups discussed in the previous section. We develop a statistical model that can be used to predict the probability of a pastoralist setting a camp at a location at a time. We denote this probability as *P*(*x*_*t*_ = *x*), where *x*_*t*_ is the location of a pastoralist at time *t*, and *x* refers to any location. We use *P*(*x*_*t*_ = *x*∣*s*_*t*_) to denote the probability of a pastoralist at location *x* at time *t* given its transhumance mode at that time, *s*_*t*_, and *P*(*s*_*t*_ = *M*_*i*_) to specify the probability of the transhumance mode at time *t* being *M*_*i*_, where *M*_*i*_ is one of the modes (M1 through M4). According to the total probability theorem [51], we can decompose *P*(*x*_*t*_ = *x*) as follows:

$$\begin{array}{c} P\left(x_{t}=x\right)=\sum_{M_{i}\in M}P\left(x_{t}=x|s_{t}=M_{i}\right)\cdot P\left(s_{t}=M_{i}\right)\;. \end{array}$$ (1)

Using the modes listed in Fig 5 and Table 1, we have *M* = {*M*_1_,*M*_2_, *M*_3_} for pastoralists in groups 1 and 2, and *M* = {*M*_1_,*M*_2_, *M*_3_, *M*_4_} for group 3. Mode M3 appears twice for all the groups, and M2 appears twice for group 3; we will discuss how to reconcile this below. The two items in Eq (1) specify two different dimensions of transhumance. We call *P*(*x*_*t*_ = *x*∣*s*_*t*_ = *M*_*i*_) a *spatial* model, and *P*(*s*_*t*_ = *M*_*i*_) a *temporal* model. Because these two dimensions are explicitly considered in our model, we will refer to this model as the spatial-temporal mobility (STM) model in the rest of this paper.

### Spatial modeling

**Fig 6:**
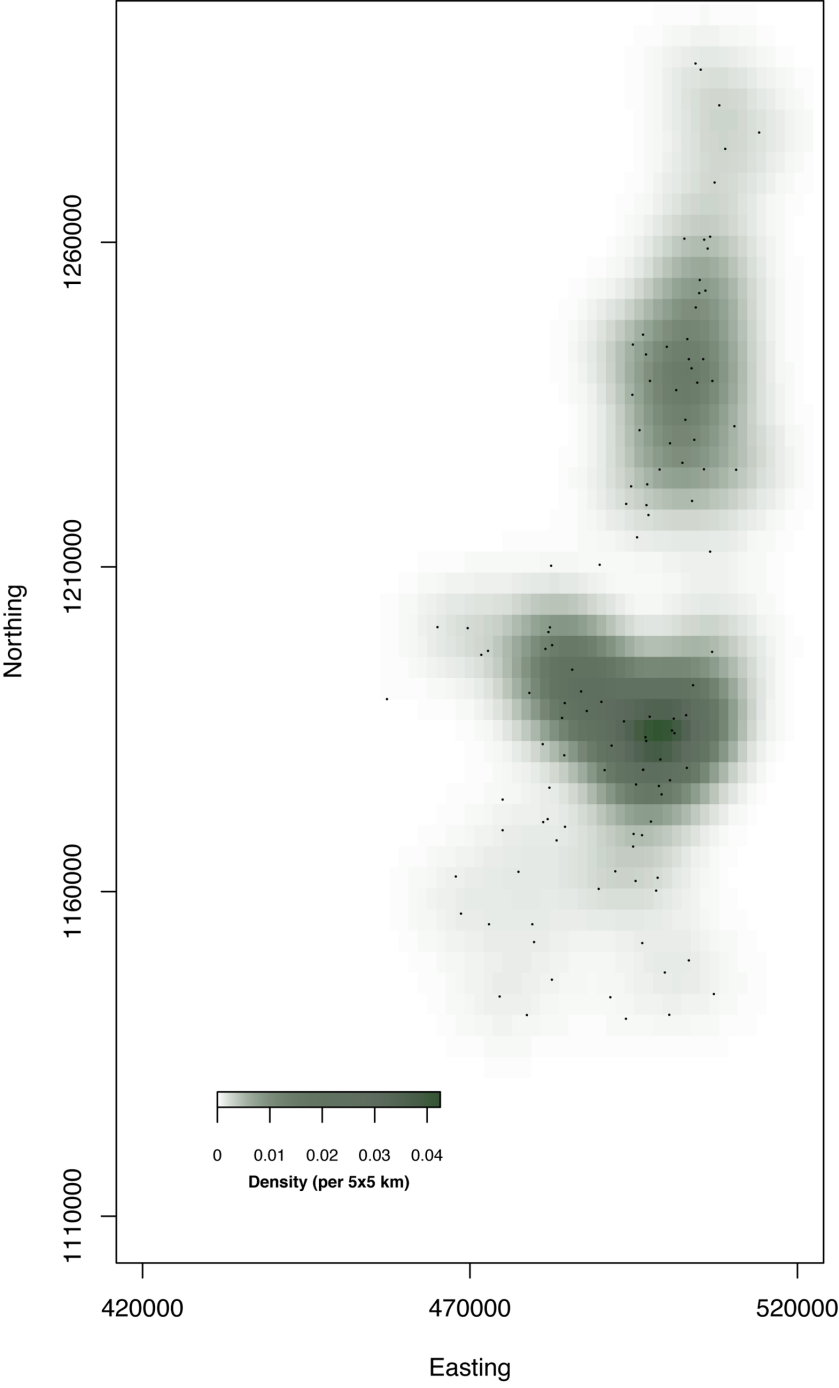
The kernel density of the camps during the hot dry season. A kernel of 5 km is used. The dots are the locations over the four years of the transhumance data.

Campsites are not randomly distributed on space. Fig 6 illustrates this point using the locations in the hot dry season for the four years in our data on a smoothed kernel density field. It is clear that pastoralists have a high tendency to go to the places that are rendered darker on the plot. For this reason, we use a bivariate normal distribution to model the locations of pastoralists, with the two variables from the X and Y coordinates on the Euclidean space.

When pastoralists are in transhumance modes M1 (all groups) and M2 (groups 1 and 2), we model their locations using a bivariate normal distribution:

$$\begin{array}{c} P\left(x_{t}=x|s_{t}=M_{i}\right)∼N\left(\mu_{i},\Sigma_{i}\right)\;, \end{array}$$ (2)

where *μ*_*i*_ and Σ_*i*_ are the mean and variance matrix of locations for transhumance mode *M*_*i*_ (*i* ∈ {1,2}).

Pastoralists in group 3 have a special case of mode M2 because the non-directional movements concentrated in two areas in this mode: the north mainly in the cold dry season and the south mainly in the hot dry season (Fig 5B). For this special case, we use a mixture of bivariate normal distributions with two components:

$$\begin{array}{c} P\left(x_{t}=x|s_{t}=M_{2}\right)=\sum_{k=1}^{2}w_{k}P_{2,k}\;, \end{array}$$ (3)

where *P*_2,*k*_ ∼ *N*(*μ*_2,*k*_, Σ_2,*k*_) is one of the two component bivariate normal distributions described by the mean (*μ*_2,*k*_) and variance matrix (Σ_2,*k*_) of locations in the north (*k* = 1) or south (*k* = 2), and *w*_*k*_ is the weight given to each distribution. Note a subscript of 2 is used to indicate the mode of M2 in this case.

In modes M3 and M4, the pastoralist moves in an overall linear fashion from an origin place to a destination. The pastoralist may move away from the linear path while following the general direction. But between the start and end points, the likelihood of the pastoralist moving away from the linear path increases initially, reaches the maximum, and then decreases to zero at the end point. We describe movements in these two modes using the Brownian bridge motion model (BBMM), a continuous time movement model with the probability of an individual being at a location related to the two end points of the path during a specified period of time from *t* = 0 to *T*, where *T* is the total number of days in M3 or M4 [45, 52]. Compared with alternative modeling approaches such as kernel estimation that often assume temporal independence, the BBMM is based on a temporal sequence of movements [53]. Thus, the expected position at time *t* follows a normal distribution:

$$\begin{array}{c} P\left(x_{t}=x|s_{t}=M_{i}\right)∼N\left(\mu_{i,t},\Sigma_{i,t}I\right)\;, \end{array}$$ (4)

where *i* is either 3 or 4 (for modes M3 and M4, respectively), *μ*_*i*,*t*_ and Σ_*i*,*t*_ are the mean and variance of locations at time *t* for mode *M*_*i*_, respectively, and ***I*** is an identity matrix. The mean location, *μ*_*i*,*t*_, is proportional to the time moving between a origin (*a*) and a destination (*b*):

$$\begin{array}{c} \mu_{i,t}=a+\left(b-a\right)\cdot t/T\;, \end{array}$$ (5)

where *T* is the total time in the mode. We discuss how these parameters are set later in the model fitting section. The variance at time *t*, Σ_*i*,*t*_, increases from 0 at *t* = 0 to the midpoint in time and then decreases to 0 when *t* = *T*:

$$\begin{array}{c} \Sigma_{i,t}=\frac{t(T-t)}{T}\Sigma_{i}\;, \end{array}$$ (6)

where Σ_*i*_ is the maximum variance related to the mobility of an individual in mode *M*_*i*_ (*i* ∈ {3,4}).

Mode M3 occurred twice for all groups (Fig 5), and we can use a subscript *k* to distinguish them as we did in the temporal model: Σ_3,1_ for the period when pastoralists move from rainy season lands to dry season lands and Σ_3,2_ when they move back. Similarly we can distinguish the mean locations for mode M3 as *μ*_3,1,*t*_ and *μ*_3,2,*t*_.

### Temporal modeling

The temporal models stipulate the probability of a particular transhumance mode at a given time. In general, we assume the likelihood of a pastoralist being in a specific mode at a given time follows a normal distribution:

$$\begin{array}{c} P\left(s_{t}=M_{i}\right)∼N\left(\tau_{i},\sigma_{i}^{2}\right) \end{array}$$ (7)

where *τ*_*i*_ and $\sigma_{i}^{2}$ are the mean and variance of dates mode *M*_*i*_. We have *M*_*i*_ to be M1 and M2 for groups 1 and 2, and M1 and M4 for group 3.

There are two cases when the same transhumance mode appears twice during the year: mode M3 for all the three groups and mode M2 for group 3 (Table 1 and Fig 5). We use the mixture of two normal distributions with two components to capture these temporal dynamics:

$$\begin{array}{c} P\left(s_{t}=M_{i}\right)=\sum_{k=1}^{2}\lambda_{k}P_{i,k}\;, \end{array}$$ (8)

where *k* (1 ≤ *k* ≤ 2) indicates the time when the mode *M*_*i*_ occurs in the year (*i* ∈ {2,3}) (for example, for M3, we have *k* = 1 for the transhumance from rainy season lands to dry season lands and *k* = 2 for the opposite direction), *P*_*i*,*k*_ follows a normal distribution $N(\tau_{i,k},\sigma_{i,k}^{2})$ where *τ*_*i*,*k*_ and $\sigma_{i,k}^{2}$ are the mean and variance of the date for mode *M*_*i*_ in component *k*, and *λ*_*k*_ is the weight given to each component.

### Reference Models

In order to compare and evaluate the above spatial-temporal mobility model, we develop two simple but non-trivial reference models. The first reference model is called KRN that is based on a two dimension kernel smoothing method [54]. Specifically, the probability of a pastoralist being at location *x* at time *t* is calculated as

$$\begin{array}{c} F\left(x_{t}=x\right)=\frac{1}{n}\sum_{i=1}^{n_{t}}K_{H}\left(x-x_{i}^{t}\right)\;, \end{array}$$ (9)

where *x* is a location determined by a pair of coordinates, *n*_*t*_ is the number of pastoralists in the data at time *t*, $x_{i}^{t}$ is the location of the *i*-th pastoralist at time *t* in the data, and *K*_*H*_(*x*) = ∣*H*∣^1/2^ *K*(*H* ^1/2^ *x*) is a kernel function that uses a 2 × 2 bandwidth matrix denoted as *H*. Function *K* satisfies ∫*K*(*x*)*dx* = 1 and we adopt a commonly used normal density form written as

$$\begin{array}{c} K\left(x\right)=\frac{1}{2\pi }e^{-\frac{1}{2}x^{T}x}\;. \end{array}$$ (10)

In other words, *K*_*H*_(*x* − *x*_*i*_) follows a normal distribution of *N*(*x*_*i*_,*H*).

The second reference model is based on a multivariate normal distribution (MVN). We utilize a bivariate normal distribution of locations for each day. Formally, the distribution of pastoralist locations at time *t* follows a normal distribution of

$$\begin{array}{c} G\left(x_{t}=x\right)∼N\left(\mu_{t},\Sigma_{t}\right)\;, \end{array}$$ (11)

where *μ*_*t*_ and Σ_*t*_ are mean and variance of locations at time *t*.

## Model Fitting

The spatial models in the STM model contain parameters denoted as *w*, *μ*, Σ, and the temporal models contain parameters denoted as *λ*, *τ* and *σ*. There are a total of 66 parameters to estimate, as summarized in Table 2. All the locations in our data set were projected to zone 33 north of the Universal Transverse Mercator coordinate system and their unit was meter. The temporal unit was day.

**Table 2:** Parameters for the spatial and temporal mobility (STM) model.

Group	Model	Transhumance Modes			
		M1	M2	M3	M4
1	Spatial	*μ*_1_	*μ*_2_	*μ*_3,1,*t*_, *μ*_3,2,*t*_	
		Σ_1_	Σ_2_	Σ_3,1_, Σ_3,2_	
	Temporal	*τ*_1_	*τ*_2_	*τ*_3,1_, *τ*_3,2_	
		$\sigma_{1}^{2}$	$\sigma_{2}^{2}$	$\sigma_{3,1}^{2}$, $\sigma_{3,2}^{2}$	
				*λ*_1_, *λ*_2_	
2	Spatial	*μ*_1_	*μ*_2_	*μ*_3,1,*t*_, *μ*_3,2,*t*_	
		Σ_1_	Σ_2_	Σ_3,1_, Σ_3,2_	
	Temporal	*τ*_1_	*τ*_2_	*τ*_3,1_, *τ*_3,2_	
		$\sigma_{1}^{2}$	$\sigma_{2}^{2}$	$\sigma_{3,1}^{2}$, $\sigma_{3,2}^{2}$	
				*λ*_1_, *λ*_2_	
3	Spatial	*μ*_1_	*μ*_2,1_, *μ*_2,2_	*μ*_3,1,*t*_, *μ*_3,2,*t*_	*μ*_4,*t*_
		Σ_1_	Σ_2,1_, Σ_2,2_	Σ_3,1_, Σ_3,2_	Σ_4_
			*w*_1_, *w*_2_		
	Temporal	*τ*_1_	*τ*_2,1_, *τ*_2,2_	*τ*_3,1_, *τ*_3,2_	*τ*_4_
		$\sigma_{1}^{2}$	$\sigma_{2,1}^{2}$, $\sigma_{2,2}^{2}$	$\sigma_{3,1}^{2}$, $\sigma_{3,2}^{2}$	$\sigma_{4}^{2}$
			*λ*_1_, *λ*_2_	*λ*_1_, *λ*_2_	

*Temporal models* were fitted using related dates extracted from the data. For the temporal models using a single normal distribution (i.e., mode M1 for all groups, mode M2 for groups 1 and 2, and mode M4 for group 3), we used the dates when pastoralists in each group moved in the corresponding mode. As discussed before, pastoralists typically stayed more than 20 days when they were in modes M1 and M2. On the other hand, pastoralists tended to stay at a location in less than 20 days when they moved directionally in modes M3 and M4. For this reason, the temporal models for mode M1 were fitted using the dates in the rainy season (days 1 through 78, and 321 through 365 in Table 1, with day 1 set on August 16) on which the pastoralists stayed for longer than 20 days. Similarly, the temporal models for mode M2 of groups 1 and 2 were fitted using the cold and hot dry seasons days (79–290) on which the pastoralists in each group stayed longer than 20 days at each location. The temporal model for pastoralists in group 3 who moved in mode M4, days of stay shorter than 20 days in the cold and hot dry seasons were used to fit the model. The actual model fitting of calculating their means and standard deviations was conducted using the maximum likelihood estimation method in an R package called mclust [55].

To fit the mixture temporal model (Eq (8)) for mode M2 for the group 3 pastoralists, dates in cold and hot dry season (day 78 through 290 in Table 1) were used if the stay is longer than 20 days. An expectation-maximization algorithm [56, 57] in the R package mclust was used to find the maximum likelihood estimation of the three sets of parameters, *λ*, *τ*, and *σ*, for the mixture of two normal distributions.

To fit the mixture model for mode M3 for all three groups, the expectation-maximization algorithm was again used to find the maximum likelihood estimation of the parameters (*λ*, *τ*, and *σ*) for the mixture of two normal distributions. As illustrated in Fig 2, the majority of the short stays (duration less than 20 days) occurred when the pastoralists were in a transit mode when they tried to migrate toward a location following a direction. For this reason, the dates for all the short stays in the year were used to fit this model.

*Spatial models* were fitted using locations related to each transhumance mode of each pastoralist group. The spatial models of mode M1 (for all groups) were fitted using the locations in the rainy season where the pastoralists stayed longer than 20 days. To fit the spatial model of M2 for groups 1 and 2, we used the set of the farthest points of each pastoralist from the rainy season locations. The mode M2 spatial model for group 3, however, was a mixture multivariate normal distribution model, and we fitted this model using the locations visited by the pastoralists in the cold and hot dry seasons.

The Brownian bridge motion variances (Eq (6)) for modes M3 and M4 were estimated using an R package called BBMM [52]. Locations and days of stay at each location when the pastoralists were in modes M3 and M4 were used to fit the spatial models for these two modes. We used the mean of variances of all pastoralists in a group as the overall variances of Σ_3,1_, Σ_3,2_, or Σ_4_. The mean locations in these models (*μ*_*i*,*t*_ in Eq (4)), however, were not directly estimated. Instead, we estimated the start and end points of each mode and used Eq (5) to compute the mean locations. We used the mean locations of the mode that immediately precedes and that follows M3 or M4 to represent the start (*a*) and end (*b*) locations, respectively, and the difference in the mean times of the preceding and following modes as the total time (*T*):

- Mode M3 appeared twice in pastoral transhumance and we used it twice separately in the spatial model. The first occurrence of mode M3 took place in the end of the rainy season for all groups 1 and 2 when the pastoralists moved toward the dry season lands and we set *a* = *μ*_1_, *b* = *μ*_2_, and *T* = *τ*_2_ − *τ*_1_, where *μ*_1_ and *μ*_2_ are the mean locations of modes M1 and M2, and *τ*_1_ and *τ*_2_ are the mean times of modes M1 and M2, respectively, for the same group. For group 3, we have *a* = *μ*_1_, *b* = *μ*_2,1_, and *T* = *τ*_2,1_ − *τ*_1_, where *μ*_2,1_ and *τ*_2,1_ are the mean location and mean time of the first component of mode M2, respectively. We can then estimate the mean location at time *t*, *μ*_3,1,*t*_, using Eq (5). For example, we have *μ*_3,1,*t*_ = *μ*_1_+(*μ*_2_ − *μ*_1_)·*t*/(*τ*_2_ − *τ*_1_) for group 1 or 2.
- The second occurrence of mode M3 was when the pastoralists move back to their rainy season lands. For groups 1 and 2 we set *a* = *μ*_2_, *b* = *μ*_1_, and *T* = 365 − (*τ*_2_ − *τ*_1_). For group 3, we used *a* = *μ*_2,2_, *b* = *μ*_1_, and *T* = 365 − (*τ*_2,2_ − *τ*_1_), where *μ*_2,2_ and *τ*_2,2_ are the second component of the mean location and mean time of mode M2, respectively, for the same group. These are input to compute *μ*_3,2,*t*_.
- To compute *μ*_4,*t*_ for mode M4 (group 3 only), we set *a* = *μ*_2,1_, *b* = *μ*_2,2_, and *T* = *τ*_2,2_ − *τ*_2,1_.

Finally, the reference models, KRN and MVN, were fitted for each day using the locations of pastoralists in each group on that day. For KRN, a bandwidth must be specified to give a standard deviation to each dimension of the reported location in the data. While the literature has indicated that 5 km is the average daily grazing distance in the area [23], we tested a wide range of bandwidths to explore the impact of bandwidth on model fitting. Specifically, we fitted the KRN model using four bandwidths: 1, 5, 10, and 20 km, and the results obtained using these bandwidths are denoted as KRN1, KRN5, KRN10, and KRN20, respectively. The R package called KernSmooth was used to fit the model [54]. The parameters of the MVN model were estimated using the mclust package [55].

## Modeling Results

**Fig 7:**
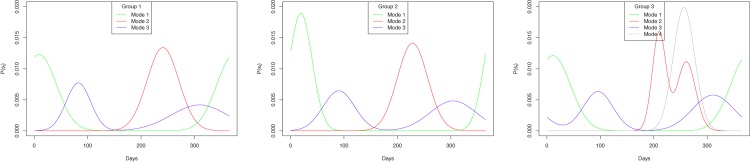
Results of fitting the temporal models. Each curve represents the probability of a particular day being in a specific transhumance mode. The horizontal axis indicates the days in a year starting on August 16.

With the four years data collected, we used 2007–2008 data to fit the models (see the result in S1 Table). The fitted models were then tested using the data from the other three years (next section). Fig 7 shows the result of fitting the temporal models. Groups 1 and 2 had similar trends in terms of how the transhumance modes changed throughout the year. Pastoralists in these groups tended to move around their rainy season locations at the beginning of the year (again, the first day here is August 16) as the peak of the curve for mode M1 concentrated on the early days (i.e., day 10 for group 1 and day 19 for group 2, see Table 2). The major transhumance mode transited to M3 when the pastoralists started their transhumance toward the dry season lands. The results suggested that M3 “took over” at around day 60 for both groups when the blue curve surpassed the green curve. After day 150 for group 1 and day 170 for group 2, the majority of the pastoralists exhibited mode M2 when they reached their dry season lands and started to stay at each location in a relatively longer period of time. This continued until day 290 (group 1) or day 280 (group 2) when the probability of mode M3 started to surpass M2 as the pastoralists began their transhumance back to the rainy season lands in the south. Mode M1 became the main transhumance mode after day 320 (group 1) or 350 (group 2) after the pastoralists arrived the south.

For group 3, while the trends related to modes M1 and M3 were still similar to those in groups 1 and 2, the curve of mode M2 now had two peaks, resulted from the mixture multivariate distribution model to match the observation that the mode M2 for this group occurs in two areas: the north and then the south of the region. It can also be noted that the new mode M4 had a significant influence between days 230 and 290 when it surpassed all the other modes. It is interesting to note that this period was also the time when all the three modes (M2, M3, and M4) were prevalent, meaning the pastoralists may not exhibit a single mode in this time. Instead, some pastoralists may stay at a location for long time (M2), some may be in the process of moving to the south (M4), and some may have started the transhumance route back to the rainy season lands (M3).

**Fig 8:**
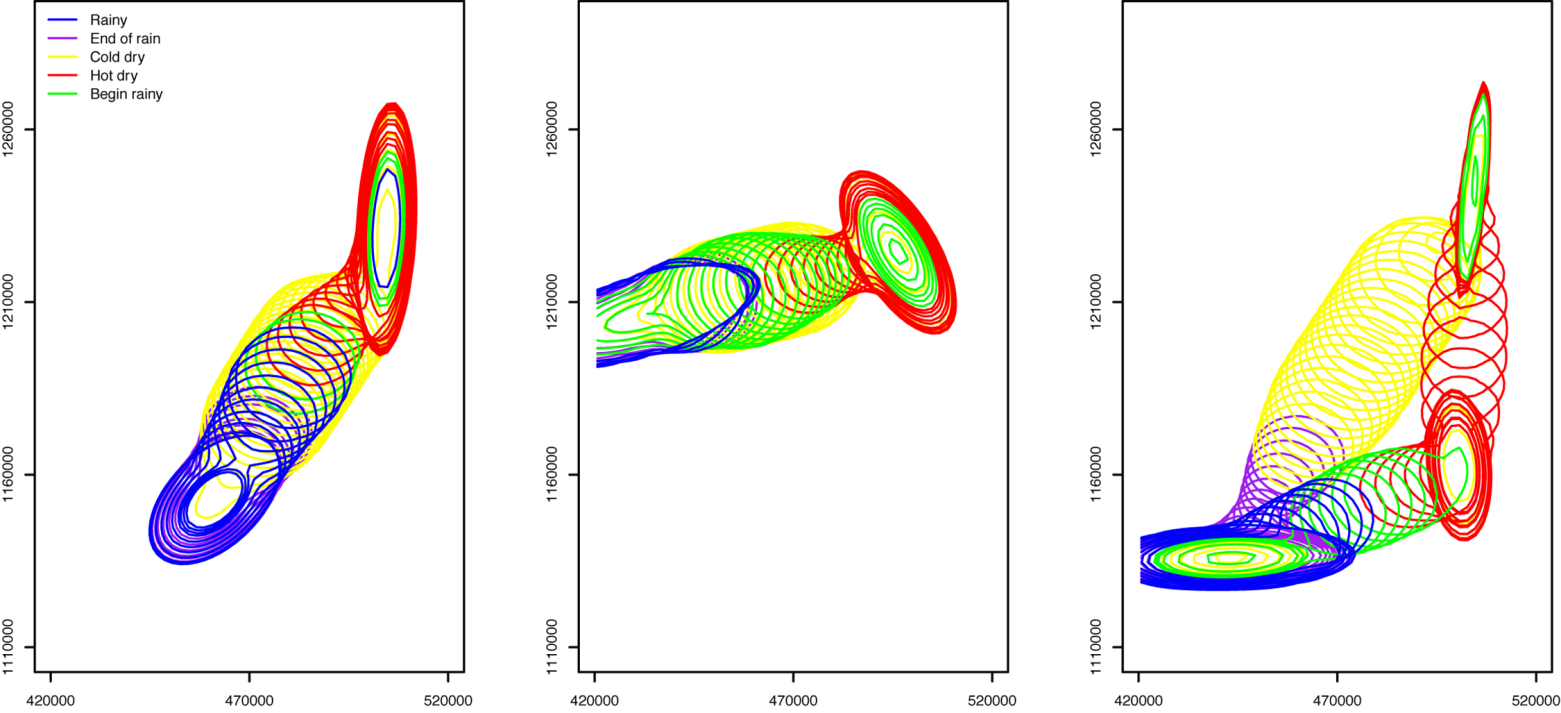
Results from the STM model. Each contour encloses an area where *P*(*x*_*t*_ = *x*) ≥ 95%. Each map shows the result after overlaying the contours of every 5 days throughout the year. Groups 1, 2, and 3 are represented in the figures from left to the right. The part of contours outside the study area is not shown in the maps.

To illustrate the STM model as a whole (Eq (1)), we discretized the continuous field of probability *P*(*x*_*t*_ = *x*) using a grid of cells with a resolution of 1 by 1 km. The center of each cell is used as the location *x* to compute the probability and for any given time *t*, we have ∑_*x*_ *P*(*x*_*t*_ = *x*) = 1. For each day, we interpolated the contour line of 95 percent probability using the grid. Areas within the contour meant that a pastoralist had a 95 percent or higher probability to be in the area. Fig 8 shows the contour lines on an interval of 5 days throughout the year for the three groups.

**Fig 9:**
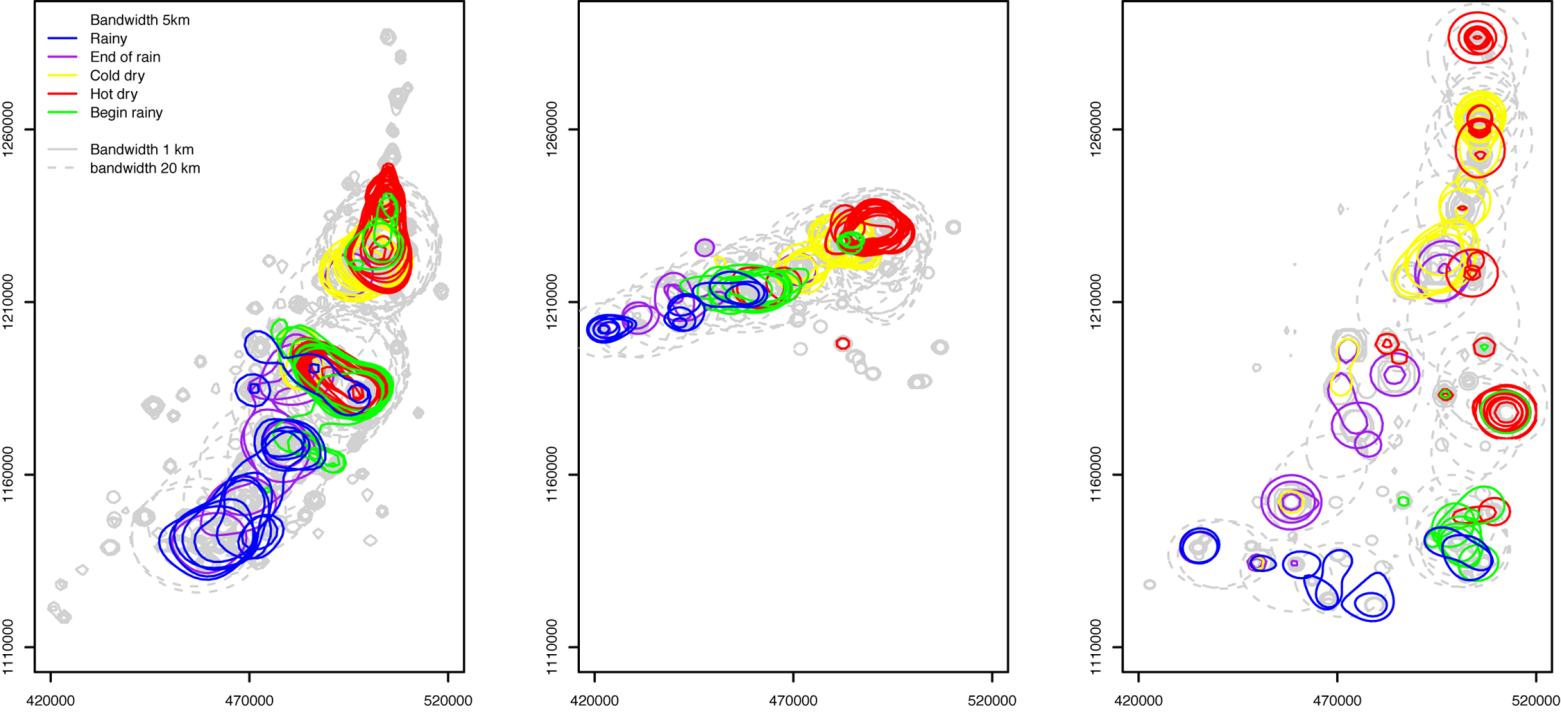
Results from the KRN model. Each contour encloses an area where *F*(*x*_*t*_ = *x*) ≥ 95%. Each map shows the result after overlaying the contours of every 5 days throughout the year. Groups 1, 2, and 3 are represented in the figures from left to the right. Each map shows the results from three bandwidths: 1 km (light grey solid lines), 5 km (colored solid lines), and 20 km (dashed light grey lines).

**Fig 10:**
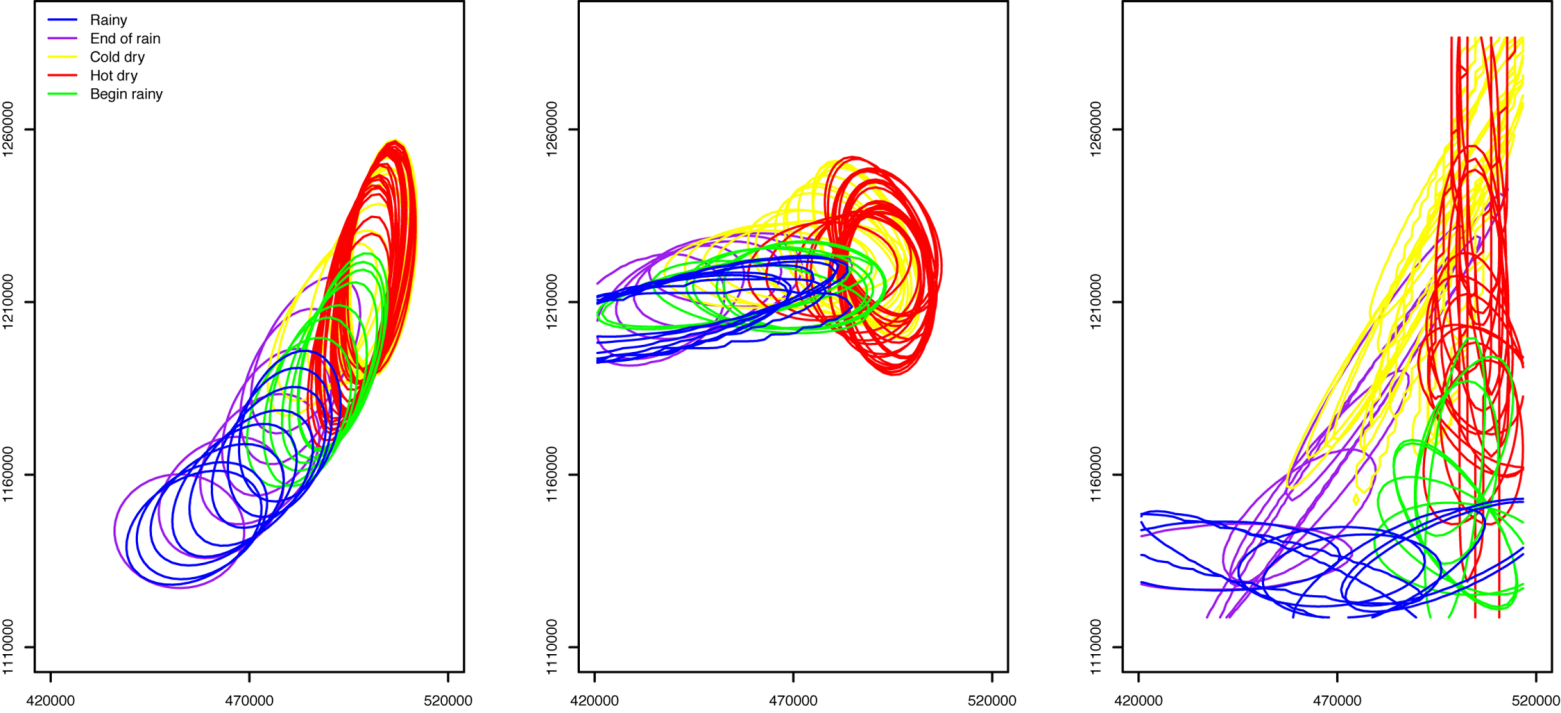
Results from the MVN model. Each contour encloses an area where *G*(*x*_*t*_ = *x*) ≥ 95%. Each map shows the result after overlaying the contours of every 5 days throughout the year. Groups 1, 2, and 3 are represented in the figures from left to the right. The part of contours outside the study area is not shown in the maps.

After parameterization of the KRN and MVN models, we again discretized their probability fields, *F*(*x*_*t*_ = *x*) and *G*(*x*_*t*_ = *x*), on the same grid as for the STM model. To illustrate these values, we again used contour lines on a 5-day interval throughout a year to represent the dynamics of pastoralist movement (Figs 9 and 10). For a time *t*, we have ∑_*x*_ *F*(*x*_*t*_ = *x*) = 1 and ∑_*x*_ *G*(*x*_*t*_ = *x*) = 1. The KRN model has four sets of results, each corresponding to a bandwidth value, and results of using three bandwidths are shown in Fig 9.

A visual observation on the contour lines in Figs 8, 9, and 10 indicates a clear difference among the three modeling approaches. Both KRN and MVN models yielded results that highly mimicked the spatial and temporal trend in the training data. Specifically, the KRN model results using bandwidths of 1 and 5 km suggested significant gaps between the contour lines (though we must consider the 5-day interval in the figure and the 95 percent cut-off value for illustration). This was because of the places in gaps were not visited by the pastoralists in the data in the given year (2007–2008). A greater bandwidth (e.g., 20 km) can be used to reduce or even eliminate the gaps. While the MVN model returned results that had similar spatial pattern as STM, MVN results appeared to be conservative in estimating the possible locations of the pastoralists. Additionally, STM results showed a smooth transition among contour lines, while the lines in MVN result tended to have abrupt changes, even for a relatively short 5-day interval.

## Model Comparison and Testing

To further evaluate the three models, we used the parameters and probabilities fitted from the 2007–2008 data (Figs 8, 9, and 10) to simulate transhumance routes and then compare these simulations with the data for all four years. Each simulated route was created using the following steps. At day *t*, we drew a sample location $x_{t}^{\prime }$ from the grid where each location in the grid was weighted by the probability from one of the fitted models. The simulated pastoralist stayed at $x_{t}^{\prime }$ for a total of *d*_*t*_ days, which was drawn from a normal distribution of $d_{t}∼N(\tau_{t}^{\prime },\sigma_{t}^{\prime 2})$, where $\tau_{t}^{\prime }$ and $\sigma_{t}^{\prime }$ are the mean and standard deviation of the days of stay by the pastoralists at time *t*. At the end of *d*_*t*_ stay, we set time to *t*+*d*_*t*_ and repeated the above process.

For each combination of the year, model, and pastoralist group, we generated 100 sets of simulations, where each set included the number of transhumance routes that was the same as the number of pastoralists in that group in our data. The three pastoralist groups in 2007–2008 were discussed above (see Fig 4). For the other three years, 2008–2009, 2009–2010, and 2010–2011, we conducted the cluster analysis to classify the pastoralists in each year’s data into three groups (again see S1 Text for details). Then the mean ($\tau_{t}^{\prime }$) and standard deviation ($\sigma_{t}^{\prime }$) of stays on each day were derived for each group and each year. For a simulated transhumance route, we measured three metrics: the average closeness between pastoralists on each day, the mean moving distance made by pastoralists between two consecutive days, and the convex hull that encloses all the locations of the route; these metrics were then used to compare the simulated routes with the data (see S2 Text for details).

Table 3 shows the mean difference in the closeness measure between the 100 simulated routes and the routes in the data for the entire year for four years. It is clear that the STM model outperformed the reference models in most of the cases as indicated by the numbers in bold typeface in the table. Specifically, the STM model yielded better measures in all but three cases. The actual difference in closeness between an STM simulation and the data varies from as low as 0.145 (group 1, 2009–2010) to 0.718 (group 3, 2010–2011). In the other three cases, two were yielded by the KRN model (1 km bandwidth) and one by the MVN. Among the KRN results using the 4 different bandwidths, the 1 km bandwidth yielded the best results in 6 cases, 5 km in 5 cases, and 10 km in 1 case.

**Table 3:** The mean difference in the closeness measure between the simulations and the data for the year. The numbers in bold indicate the best measure among the three models for each year.

Metric	Group	Model	2007–2008	2008–2009	2009–2010	2010–2011
Closeness	1	STM	**0.182**	**0.238**	**0.145**	0.272
		KRN1	0.185	0.240	0.218	**0.174**
		KRN5	0.195	0.304	0.230	0.218
		KRN10	0.268	0.256	0.301	0.277
		KRN20	0.589	0.516	0.599	0.626
		MVN	0.206	0.345	0.232	0.234
	2	STM	**0.385**	**0.600**	0.450	**0.395**
		KRN1	0.415	0.761	**0.390**	0.752
		KRN5	0.598	0.852	0.695	0.496
		KRN10	0.508	0.723	0.528	0.835
		KRN20	1.173	1.338	1.104	1.511
		MVN	0.600	0.844	0.714	0.510
	3	STM	0.359	**0.366**	**0.364**	**0.718**
		KRN1	0.499	0.675	0.780	0.973
		KRN5	0.348	0.553	0.392	0.760
		KRN10	0.630	0.563	0.769	0.930
		KRN20	0.959	0.512	1.121	1.175
		MVN	**0.265**	0.555	0.423	0.814

**Fig 11:**
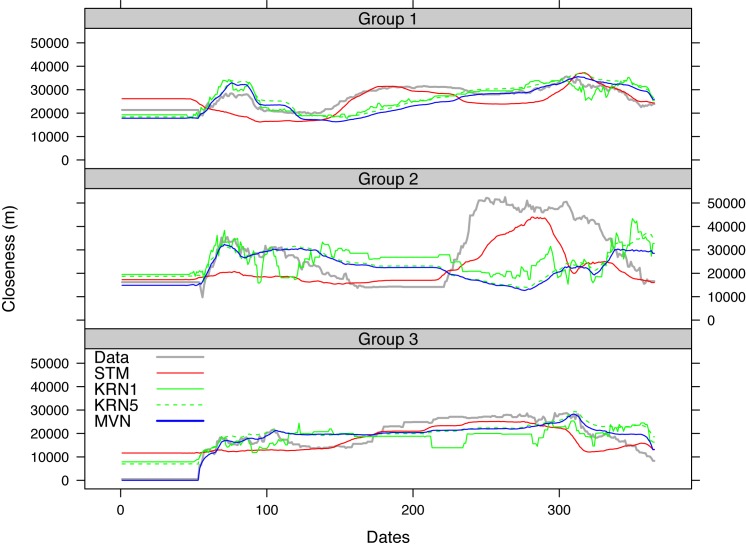
Daily mean closeness between pastoralists obtained from the 2007–2008 data and 100 simulations using STM, KRN, and MVN.

Fig 11 shows the daily closeness measure between pastoralists in the 2007–2008 data and the averaged daily closeness of the 100 simulations from the three models. The difference in KRN results using 1 and 5 km bandwidths was relatively small when compared with the difference between STM results and the reference models. The KRN and MVN models generally captured the closeness between pastoralists when they were located in the rainy season lands. This is the period of time when all the pastoralists stayed in their rainy season locations and tended not to move as frequently as in other seasons (because of abundant forage due to seasonal rainfall). But the red curves (STM) started to be closer to the data curve approximately after day 120. This is the time when the pastoralists had moved out of the rainy season lands, and they exhibited a diversity in their spatial movements as some of the pastoralists had arrived in the dry season lands, while other were still on their transhumance paths toward dry season destinations. The STM model appeared to capture such diversity and dynamics.

Table 4 shows the mean difference of the moving distance between the 100 simulated routes and the routes in the data for the whole year. It is clear that the STM model outperformed the other two models in all of the cases as indicated by the numbers in bold typeface. The difference in moving distance yielded by all the STM simulations ranges from 1.702 (group 1, 2009–1020) to 4.465 (group 3, 2010–2011) times than the moving distance in the data, and the smoothed moving distance measures for the STM model were all below 1.0 except in group 1 for year 2010–2011. Given the diversity of pastoral movements, it is perhaps unlikely for any model to predict exactly the same as in the data. The smoothed measures indicate the effectiveness of the models in average.

**Table 4:** The mean difference in the moving distance measure between the simulations and the data for the year. The numbers in bold indicate the best measure among the three models for each year.

Metric	Group	Model	2007–2008	2008–2009	2009–2010	2010–2011
Moving distance	1	STM	**2.118**	**1.702**	**1.707**	**2.323**
		KRN1	2.265	1.985	1.985	2.215
		KRN5	2.297	1.901	1.971	2.586
		KRN10	2.641	2.432	2.177	2.598
		KRN20	3.694	3.356	2.925	3.663
		MVN	2.181	1.804	1.903	2.462
	2	STM	**2.512**	**2.709**	**2.486**	**1.838**
		KRN1	2.962	3.804	3.924	5.263
		KRN5	2.767	3.168	2.678	2.273
		KRN10	3.626	4.911	4.610	6.346
		KRN20	5.450	7.159	6.250	8.845
		MVN	2.575	3.011	2.548	2.136
	3	STM	**2.367**	**3.122**	**3.301**	**4.465**
		KRN1	3.799	3.413	3.840	4.809
		KRN5	3.076	4.037	3.907	5.482
		KRN10	4.254	3.394	3.989	5.018
		KRN20	5.363	4.383	4.871	6.013
		MVN	2.888	3.748	3.782	5.146
Smoothed moving distance	1	STM	**0.916**	**0.818**	**0.677**	**1.065**
		KRN1	1.145	1.114	0.990	1.247
		KRN5	1.133	1.080	0.995	1.355
		KRN10	1.585	1.508	1.186	1.619
		KRN20	2.512	2.415	1.874	2.533
		MVN	1.043	0.989	0.933	1.250
	2	STM	**0.935**	**0.506**	**0.389**	**0.468**
		KRN1	1.013	1.348	1.258	1.028
		KRN5	1.199	1.056	0.748	0.806
		KRN10	1.681	2.168	1.604	1.819
		KRN20	2.936	3.648	2.551	2.821
		MVN	1.080	1.002	0.743	0.753
	3	STM	**0.450**	**0.743**	**0.769**	**0.782**
		KRN1	1.327	1.287	1.195	1.377
		KRN5	1.099	1.512	1.209	1.197
		KRN10	1.629	1.323	1.191	1.559
		KRN20	2.536	1.994	1.707	2.257
		MVN	0.930	1.298	1.217	1.104

**Fig 12:**
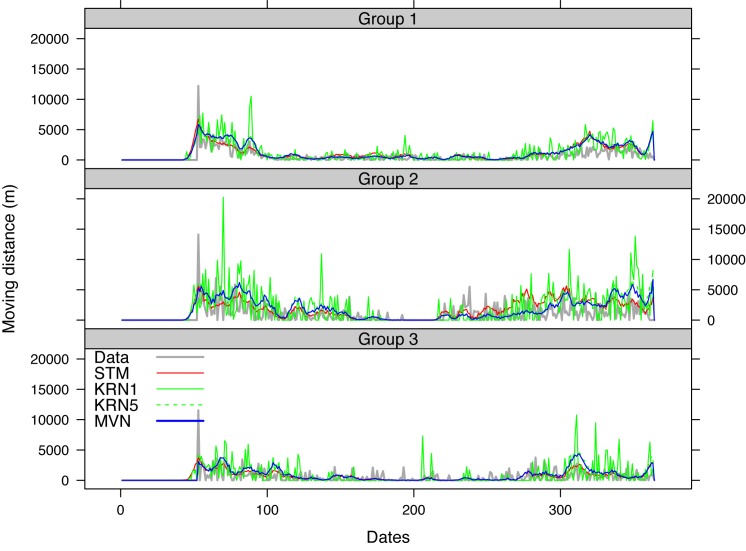
Daily mean moving distance obtained from the 2007–2008 data and 100 simulations using STM, KRN, and MVN.

Fig 12 shows the dynamics of the mean daily moving distance over the 100 simulations for all the groups against the 2007–2008 data. The Data curves in this figure show the diversity of the pastoralist movements as these curves dramatically change through time, caused by the diverse movement pattern of different pastoralists. The pastoralists generally do not move at the same time, except at the end of the rainy season when they have the pressure to move toward the dry season lands in the north. Therefore daily moving distance greatly varies between pastoralists and from day to day as some may stay in a camp for a long time while others are on the move. To show the trend more effectively, we used a 20-day moving window to smooth the moving distance measures (see S1 Fig) and the smoothing result suggested that all the three models captured the overall trend of moving distance exhibited in the data and it appeared that the STM curve stayed closer to the data curve for most of the days.

**Fig 13:**
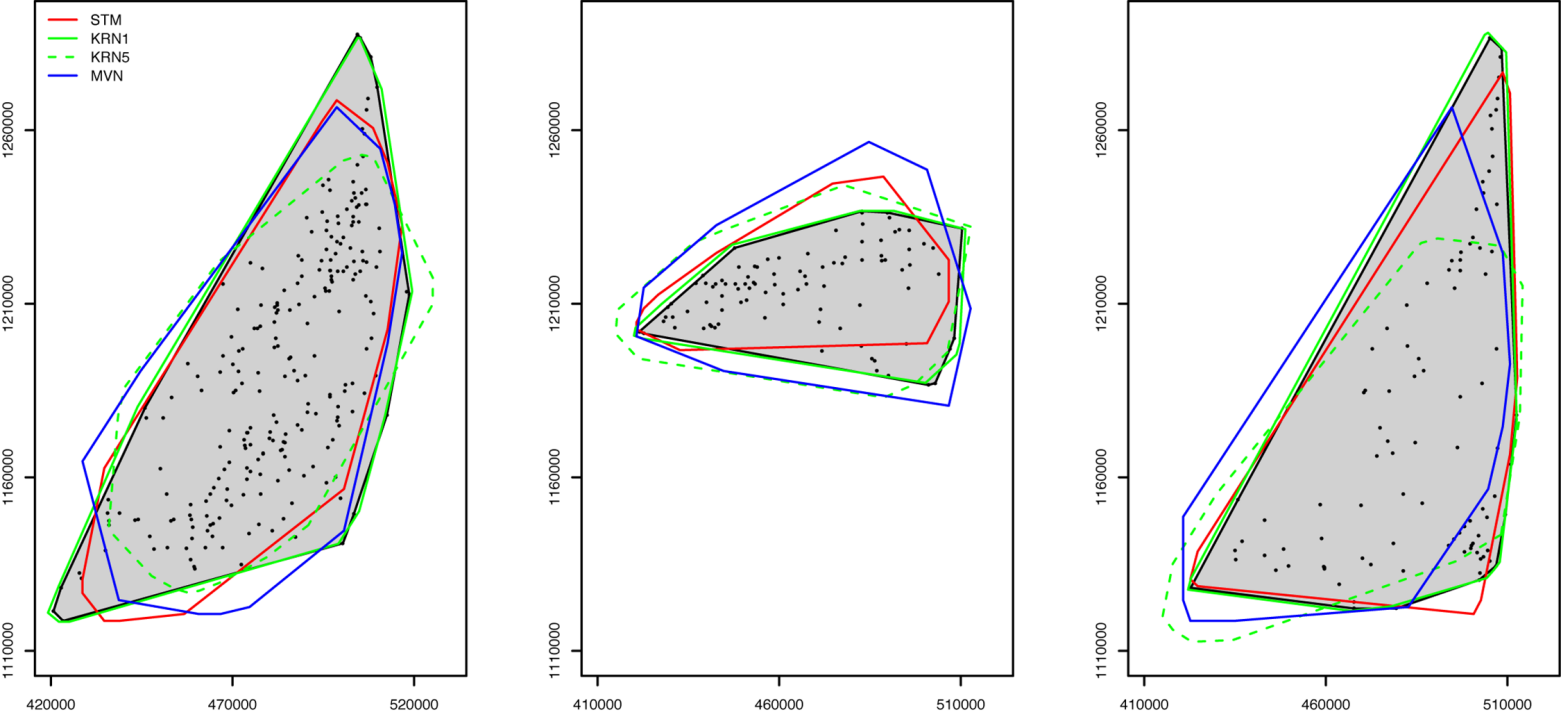
The convex hulls of the data (shaded) and of simulation results for year 2007–2008. The dots represent the locations of the pastoralists in the survey data. Pastoralist groups 1 through 3 are represented in the left, center, and right maps, respectively. For each group, only one example of the 100 simulations is shown.

Due to the variation of convex hulls in different simulations, it is difficult to visualize all the hulls in one figure. Here we show some example convex hulls from the simulations using the models along with that from the data for the three pastoralist groups (Fig 13). It appeared that the simulated pastoralist locations using the three models were generally consistent with the convex hulls from the data, and it is clear that the KRN results of 1 km bandwidth match the data for most of the cases because the areas enclosed by the solid green lines (1 km KRN results) closely match the grey areas (data). Table 5 includes the overlapped convex hull area ratio between each of the modeling results and the data.

**Table 5:** The mean difference in overlapped convex hull ratio between the simulations and the data for the year. The numbers in bold indicate the best measure among the three models for each year. Italic numbers refer to the highest ratios in all models except KRN1. The rows marked as DATA show the overlapped convex hull ratio between the data of the year and that of year 2007–2008 for each group.

Metric	Group	Model	2007–2008	2008–2009	2009–2010	2010–2011
Overlapped area ratio	1	STM	*0.863*	0.815	0.822	*0.838*
		KRN1	**0.972**	**0.872**	**0.841**	**0.900**
		KRN5	0.858	0.794	0.817	0.790
		KRN10	0.734	*0.834*	*0.841*	0.710
		KRN20	0.525	0.612	0.625	0.502
		MVN	0.841	0.815	0.837	0.778
		DATA	1.000	0.974	0.999	0.913
	2	STM	*0.911*	0.817	***0.802***	***0.854***
		KRN1	**0.937**	0.791	0.689	0.664
		KRN5	0.796	0.788	0.715	0.783
		KRN10	0.687	0.812	0.679	0.668
		KRN20	0.444	0.562	0.551	0.553
		MVN	0.847	***0.826***	0.759	0.814
		DATA	1.000	0.935	0.816	0.835
	3	STM	0.801	0.743	0.673	0.594
		KRN1	**0.976**	**0.957**	**0.911**	**0.903**
		KRN5	*0.820*	*0.844*	0.711	0.689
		KRN10	0.767	0.751	*0.738*	*0.786*
		KRN20	0.550	0.551	0.567	0.612
		MVN	0.785	0.808	0.726	0.660
		DATA	1.000	0.979	0.913	0.911

The close match in convex hulls between the 1 km KRN model result and the data, however, may not be an indication that the 1 km bandwidth KRN outperforms all other models. Using a 1km bandwidth, the kernel smoothing model produced a field where the high density areas were extremely close to the original data points as indicated by the very small contours in solid grey lines for the 1 km bandwidth in Fig 11. In this way, the simulation process essentially sampled the original points which led to a close match between the convex hulls of the simulation (1km bandwidth) and the data. However, we should note that the overlapped convex hull area ratios for KRN1 (1 km bandwidth) tend to be high for the first year (used for data fitting) and then decrease significantly in the other years. This is because the pastoralists do not always use the same set of campsites over the years, as shown by the overlap ratios between the convex hulls of the data between other years and the first year (i.e., the numbers in the DATA rows in Table 5 are not 1.000 except for the first year itself). This is especially the case for group 2 where the ratio for KRN1 decreases from 0.937 for the first year to 0.664 for the fourth year. It should be noted that group 2 also showed the least overlapped area of the convex hulls from the data, meaning the pastoralists had a relatively large variation of their annual paths through the four years. In the last two years for this group, the STM actually outperformed the other models in terms of the overlapped area. Putting aside the KRN results with 1 km bandwidth, the STM model outperformed the other models in 5 out of the 12 cases as indicated by the italic numbers in Table 5, and the KRN5, KRN10, and MVN models outperformed in 2, 4, and 1 cases out of 12, respectively.

We then tested whether STM statistically had the same mean measure as either KRN or MVN. Given the closeness of 1 km bandwidth KRN results to the original training data, we only tested the STM, KRN (5 km), and MVN models. We ran one-tailed *t* tests with an alternative hypothesis stating that, based on the 100 simulations for each year, the mean of STM results were significantly better (i.e., smaller for moving distance and closeness, and greater for overlapped convex area ratio) than those of the other two models for each a given metric. The results (S2 Table) show that, except for the few cases that are italicized, the STM results were significantly better than results from other models (at a significance level of 0.05), meaning STM yielded simulations that were closer to the data than the results from the KRN5 and MVN models.

The test results were also consistent with the results in Table 3, where the bold numbers associated with STM were also significant in the *t* tests. The only exception is the overlapped area ratio in 2007–2008 for group 1 where STM was greater (better) than that of the KRN model, but the test did not show significant difference between them (with a p-value of 0.067). For all four years, the test results indicated that STM returned significantly smaller closeness measure, except for the third group in year 2007–2008 and the first group in year 2010–2011. For the moving distance measure, results from the STM model are significantly closer to the data than the other models. The tests on overlapped area ratio, however, did not suggest a clear trend. In a half of the cases STM showed a significant larger overlapped area with the data, meaning STM results have a higher overlapped area with the data than the other models, but no significant difference was reported for the other half of the cases.

## Discussion

An important effort in mobility research has been steered toward finding fundamental governing rules in movements and mobility is often represented using a measure of movement step length. Much research has been conducted to develop models to explain the probability distribution of such length [58–61]. Using this type of models, regularity of human mobility has been found in different kinds of data sets such as mobile phone users, taxicabs on street networks, and social media check-ins [62–64]. While this line of research sheds insightful light to our understanding of how humans (and animals) move, mobility can be more directly related to the spatial and temporal dimensions of movements. In our model, we represent these two dimensions explicitly by estimating of the probability of an individual at a location at a time (Eq (1)). For this reason, we name our model the spatial and temporal mobility (STM) model. The STM model integrates the locational distribution of the pastoralists with respect to their transhumance movement modes and the temporal distribution of each mode throughout the year. The integration of the two dimensions enables the STM model to capture the seasonality and hence the environmental drives that result in the annual cycles in the transhumance pattern in our study region. The two reference models, on the other hand, are developed in a “faithful” fashion to the data because the parameters are fitted for every day from the data, and, in this way, the temporal trend is implicit and separated from the process.

To build the STM model, we do not assume that the probability, *P*(*x*_*t*_ = *x*), follows a particular statistical distribution. Instead, the total probability can be decomposed into the sum of a number of mobility modes and we can fit the model based on the identification of the modes in the data. The key to applying the STM model to other data is to identify the unique movement modes that drive different types of spatial and temporal behaviors because of different processes. Individuals’ daily activities, for example, are significantly different between weekdays and weekends, and between day and night [40, 64]. Animal populations also have migratory and stationary periods in an annual cycle [65]. Though the exact triggers between modes are problem specific and may be difficult to identify, the modes themselves can be described and modeled. Successfully identifying these modes will help develop the spatial and temporal models in the STM model, and retrieve appropriate data to fit the model. While the focus of this paper is to demonstrate the development of the STM model using the transhumance data in the Far North Region of Cameroon, the modeling framework can be extended to other phenomena such as movements on a network, as well as space-time pattern of individual activities such as studied in the time geography literature [41, 66].

The next step of this particular research is to incorporate the STM model with models that can be used to describe the space-time distribution of other cattle herds (e.g., sedentary farmers) in the region and to investigate how the contact between these two groups of population changes throughout the year and how that may impact disease transmission and other social/economic issues. In this paper we focused on modeling the transhumance patterns of mobile pastoralists in the Far North Region. However, mobile pastoralists are not living in isolation; they live interspersed with agricultural populations that do not engage in seasonal movements. To understand the potential role of pastoral mobility in the transmission of infectious diseases, it is just as critical to examine what the role is of neighboring populations that do not make seasonal movements, which is what we plan to do in our future studies. Research in such a direction will also contribute to the recent developments in the research efforts on human mobility, public health, and infectious diseases transmission [65, 67–69].

## Supporting Information

S1 Text Classification of transhumant routes. (PDF)

S2 Text Model comparison metrics. (PDF)

S1 Fig Smoothed daily mean moving distance obtained from the 2007–2008 data and 100 simulations using STM, KRN, and MVN. (PDF)

S1 Table Parameters estimated for the spatial and temporal models in the (STM). (PDF)

S2 Table Results of *t* tests on STM versus the two reference models. (PDF)

## References

[1] Tatem AJ, Hay SI, Rogers DJ. Global traffic and disease vector dispersal. Proceedings of the National Academy of Sciences. 2006;103(16):6242–6247. 10.1073/pnas.0508391103 PMC1435368 16606847

[2] Keeling MJ, Rohani P. Modeling infectious diseases in humans and animals Princeton, NJ: Princeton University Press; 2008.

[3] Stoddard ST, Morrison AC, Vazquez-Prokopec GM, Paz Soldan V, Kochel TJ, Kitron U, et al The Role of Human Movement in the Transmission of Vector-Borne Pathogens. PLOS Neglected Tropical Diseases. 2009;3(7):e481 10.1371/journal.pntd.0000481 19621090 PMC2710008

[4] Rivas AL, Fasina FO, Hoogesteyn AL, Konah SN, Febles JL, Perkins DJ, et al Connecting network properties of rapidly disseminating epizoonotics. PLoS ONE. 2012;7(6):e39778 10.1371/journal.pone.0039778 22761900 PMC3382573

[5] Wang Q, Taylor JE. Quantifying human mobility perturbation and resilience in Hurricane Sandy. PLoS ONE. 2014;9(11):e112608 10.1371/journal.pone.0112608 25409009 PMC4237337

[6] Stenning DJ. Transhumance, migratory drift, migration; patterns of pastoral Fulani nomadism. Journal of the Anthropological Institute of Great Britain and Ireland. 1957;87(1):57–73. 10.2307/2843971

[7] Barth F. Nomads of South Persia: the Basseri tribe of the Khamseh confederacy. Boston, MA: Little, Brown and Company; 1961.

[8] Behnke RH, Fernandez-Gimenez ME, Turner MD, Stammler F. Pastoral migration: mobile systems of livestock husbandry In: Milner-Gulland EJ, Fryxell JM, Sinclair ARE, editors. Animal Migration: A Synthesis. Oxford: Oxford University Press; 2011 p. 144–171.

[9] Pyke GH. Optimal foraging theory: a critical review. Annual Review of Ecology and Systematics. 1984;15:523–575. 10.1146/annurev.es.15.110184.002515

[10] Moritz M, Hamilton IM, Chen YJ, Scholte P. Mobile pastoralists in the Logone Floodplain distribute themselves in an Ideal Free Distribution. Current Anthropology. 2014;55:115–122. 10.1086/674717

[11] Spergser J, Macher K, Kargl M, Lysnyansky I, Rosengarten R. Emergence, re-emergence, spread and host species crossing of Mycoplasma bovis in the Austrian Alps caused by a single endemic strain. Veterinary Microbiology. 2013;164(3–4):299–306. 10.1016/j.vetmic.2013.02.007 23490560

[12] Mazeri S, Scolamacchia F, Handel IG, Morgan KL, Tanya VN, Barend M. Risk factor analysis for antibodies to Brucella, Leptospira and C. burnetii among cattle in the Adamawa Region of Cameroon: A cross-sectional study. Tropical Animal Health and Production. 2012;45(2):617–623. 10.1007/s11250-012-0268-0 23117621

[13] Chevalier V, Mondet B, Diaite A, Lancelot R, Fall AG, Ponçon N. Exposure of sheep to mosquito bites: Possible consequences for the transmission risk of Rift Valley Fever in Senegal. Medical and Veterinary Entomology. 2004;18(3):247–255. 10.1111/j.0269-283X.2004.00511.x 15347392

[14] Msami HM, Ponela-Mlelwa T, Mtei BJ, Kapaga AM. Contagious bovine pleuropneumonia in Tanzania: Current status. Tropical Animal Health and Production. 2001;33(1):21–28. 10.1023/A:1010377325566 11234189

[15] Macpherson CNL. The effect of transhumance on the epidemiology of animal diseases. Preventive Veterinary Medicine. 1995;25(2):213–224. 10.1016/0167-5877(95)00539-0

[16] Zhou H, Chai S, Craig P, Delattre P, Quere J, Raoul F, et al Epidemiology of alveolar echinococcosis in Xinjiang Uygur autonomous region, China: a preliminary analysis. Annals of Tropical Medicine and Parasitology. 2000;94(7):715–729. 11144813 10.1080/00034983.2000.11813595

[17] Aidaros HA. Regional status and approaches to control and eradication of foot and mouth disease in the Middle East and North Africa. OIE Revue Scientifique et Technique. 2002;21(3):451–458. 10.20506/rst.21.3.1348 12523686

[18] Peters PE. Inequality and social conflict over land in Africa. Journal of Agrarian Change. 2004;4(3):269–314. 10.1111/j.1471-0366.2004.00080.x

[19] Perry B, Rich K. Poverty impacts of foot-and-mouth disease and the poverty reduction implications of its control. Veterinary Record. 2007;160(7):238–241. 10.1136/vr.160.7.238 17308024

[20] Aune K, Rhyan JC, Russell R, Roffe TJ, Corso B. Environmental persistence of Brucella abortus in the Greater Yellowstone Area. Journal of Wildlife Management. 2012;76(2):253–261. 10.1002/jwmg.274

[21] Rebaudet S, Sudre B, Faucher B, Piarroux R. Environmental determinants of cholera outbreaks in inland Africa: A systematic review of main transmission foci and propagation routes. Journal of Infectious Diseases. 2013;208(SUPPL. 1):S46–S54. 10.1093/infdis/jit195 24101645

[22] De Boer W, Prins H. Decisions of cattle herdsmen in Burkina Faso and optimal foraging models. Human ecology. 1989;17(4):445–464. 10.1007/BF00889500

[23] Moritz M, Soma E, Scholte P, Xiao N, Taylor L, Juran T, et al An Integrated Approach to Modeling Grazing Pressure in Pastoral Systems: The Case of the Logone Floodplain (Cameroon). Human Ecology. 2010;38:775–789. 10.1007/s10745-010-9361-z

[24] Schareika N. Know to move, move to know: ecological knowledge and herd movement strategies among the Wodaabe of Southeastern Niger. Rome: FAO Inter-Departmental Working Group on Biological Diversity for Food and Agriculture; 2003.

[25] Boutrais J. Hautes terres d’élevage au Cameroun. Paris: ORSTOM Éditions; 1996.

[26] Bassett TJ, Turner MD. Sudden shift or migratory drift? Fulbe herd movements to the Sudano-Guinean region of West Africa. Human Ecology. 2007;35:33–49. 10.1007/s10745-006-9067-4

[27] Coughenour MB, Ellis JE, Swift DM, Coppock DL, Galvin K, McCabe JT, et al Energy extraction and use in a nomadic pastoral ecosystem. Science. 1985;230(4726):619–625. 10.1126/science.230.4726.619 17797276

[28] Ellis JE, Swift DM. Stability of African pastoral ecosystems: alternate paradigms and implications for development. Journal of Range Management Archives. 1988;41(6):450–459. 10.2307/3899515

[29] McCabe JT. Cattle Bring Us to Our Enemies: Turkana Ecology, Politics, and Raiding in a Disequilibrium System Ann Arbor, MI: Michigan University Press; 2004.

[30] Brottem L, Turner MD, Butt B, Singh A. Biophysical Variability and Pastoral Rights to Resources: West African Transhumance Revisited. Human Ecology. 2014;42(3):351–365. 10.1007/s10745-014-9640-1

[31] Coppolillo PB. The Landscape Ecology of Pastoral Herding: Spatial Analysis of Land Use and Livestock Production in East Africa. Human Ecology. 2000;28(4):527–560. 10.1023/A:1026435714109

[32] Butt B. Seasonal space-time dynamics of cattle behavior and mobility among Maasai pastoralists in semi-arid Kenya. Journal of Arid Environments. 2009;74:403–413. 10.1016/j.jaridenv.2009.09.025

[33] Anderson DM, Estell RE, Cibils AF. Spatiotemporal Cattle Data—A Plea for Protocol Standardization. Positioning. 2013;4:115–136. 10.4236/pos.2013.41012

[34] Adriansen HK, Nielsen TT. Going where the grass is greener: on the study of pastoral mobility in Ferlo, Senegal. Human Ecology. 2002;30:215–227. 10.1023/A:1015692730088

[35] Galaty JG. The indigenisation of pastoral modernity: territoriality, mobility, and poverty in Dryland Africa In: Bollig M, Schnegg M, Wotzka HP, editors. Pastoralism in Africa: past, present, and future. New York: Berghahn; 2013 p. 473–510.

[36] Vosloo W, Bastos A, Sangare O, Hargreaves S, Thomson G. Review of the status and control of foot and mouth disease in sub-Saharan Africa. Revue Scientifique Et Technique-Office International Des Epizooties. 2002;21(3):437–445. 10.20506/rst.21.3.1349 12523685

[37] Bronsvoort BdC, Nfon C, Hamman S, Tanya V, Kitching R, Morgan K. Risk factors for herdsman-reported foot-and-mouth disease in the Adamawa Province of Cameroon. Preventive Veterinary Medicine. 2004;66(1):127–139. 10.1016/j.prevetmed.2004.09.010 15579340

[38] Hägerstrand T. What about people in regional science? Papers of the Regional Science Association. 1970;24:7–21.

[39] Miller H. Modelling accessibility using space-time prism concepts within geographical information systems. International Journal of Geographical Information System. 1991;5(3):287–301. 10.1080/02693799108927856

[40] Kwan MP. Space-time and integral measures of individual accessibility: a comparative analysis using a point-based framework. Geographical Analysis. 1998;30(3):191–216. 10.1111/j.1538-4632.1998.tb00396.x

[41] Miller H. A measurement theory for time geography. Geographical Analysis. 2005;37(1):17–45. 10.1111/j.1538-4632.2005.00575.x

[42] Worton BJ. Kernel methods for estimating the utilization distribution in home-range studies. Ecology. 1989;70(1):164–168. 10.2307/1938423

[43] Winter S, Yin ZC. Directed movements in probabilistic time geography. International Journal of Geographical Information Science. 2010;24(9):1349–1365. 10.1080/13658811003619150

[44] Downs JA. Time-Geographic Density Estimation for Moving Point Objects In: Fabrikant S, Reichenbacher T, van Kreveld M, Schlieder C, editors. Geographic Information Science. vol. 6292 of Lecture Notes in Computer Science. Springer Berlin Heidelberg; 2010 p. 16–26.

[45] Horne JS, Garton EO, Krone SM, Lewis JS. Analyzing animal movements using Brownian bridges. Ecology. 2007;88:2354–2363. 10.1890/06-0957.1 17918412

[46] Seignobos C, Iyébi-Mandjek O, editors. Atlas de la Province Extréme-Nord Cameroun. Paris: IRD & MINREST; 2000.

[47] Scholte P, Kari S, Moritz M, Prins H. Pastoralist Responses to Floodplain Rehabilitation in Northern Cameroon. Human Ecology. 2006;34:27–51. 10.1007/s10745-005-9001-1

[48] Moritz M, Scholte P, Hamilton IM, Kari S. Open Access, Open Systems: Pastoral Management of Common-Pool Resources in the Chad Basin. Human Ecology. 2013;41:351–365. 10.1007/s10745-012-9550-z

[49] Levins R. The strategy of model building in population biology. American scientist. 1966;54(4):421–431.

[50] Giere RN. How models are used to represent reality. Philosophy of science. 2004;71(5):742–752. 10.1086/425063

[51] Papoulis A. Probability, Random Variables, and Stochastic Processes. 2nd ed New York: McGraw-Hill; 1984.

[52] Sawyer H, Kauffman MJ, Nielson RM, Horne JS. Identifying and prioritizing ungulate migration routes for landscape-level conservation. Ecological Applications. 2009;19:2016–2025. 10.1890/08-2034.1 20014575

[53] Worton BJ. A review of models of home range for animal movement. Ecological Modeling. 1987;38(6):277–298. 10.1016/0304-3800(87)90101-3

[54] Wand MP, Jones MC. Kernel Smoothing. London: Chapman & Hall; 1995.

[55] Fraley C, Raftery AE, Murphy TB, Scrucca L. mclust Version 4 for R: Normal Mixture Modeling for Model-Based Clustering, Classification, and Density Estimation. Department of Statistics, University of Washington; 2012.

[56] Dempster AP, Laird NM, Rubin DB. Maximum likelihood from incomplete data via the EM algorithm. Journal of the Royal Statistical Society Series B (Methodological). 1977;39(1):1–38.

[57] McLachlan GJ, Peel D. Finite Mixture Models. New York: John Wiley & Sons; 2000.

[58] Barabasi AL. The origin of bursts and heavy tails in human dynamics. Nature. 2005;435(7039):207–211. 10.1038/nature03459 15889093

[59] Brockmann D, Hufnagel L, Geisel T. The scaling laws of human travel. Nature. 2006;439(7075):462–465. 10.1038/nature04292 16437114

[60] Brown CT, Liebovitch LS, Glendon R. Lévy flights in Dobe Ju/’hoansi foraging patterns. Human Ecology. 2007;35(1):129–138. 10.1007/s10745-006-9083-4

[61] Gonzalez MC, Hidalgo CA, Barabasi AL. Understanding individual human mobility patterns. Nature. 2008 06;453(7196):779–782. 10.1038/nature06958 18528393

[62] Jiang B, Yin J, Zhao S. Characterizing the human mobility pattern in a large street network. Physical Review E. 2009;80(2):021136 10.1103/PhysRevE.80.021136 19792106

[63] Song C, Qu Z, Blumm N, Barabási AL. Limits of predictability in human mobility. Science. 2010;327(5968):1018–1021. 10.1126/science.1177170 20167789

[64] Cho E, Myers SA, Leskovec J. Friendship and Mobility: User Movement in Location-based Social Networks In: Proceedings of the 17th ACM SIGKDD International Conference on Knowledge Discovery and Data Mining. KDD’11. New York, NY, USA: ACM; 2011 p. 1082–1090.

[65] Tøttrup AP, Klaassen RH, Strandberg R, Thorup K, Kristensen MW, Jørgensen PS, et al The annual cycle of a trans-equatorial Eurasian–African passerine migrant: different spatio-temporal strategies for autumn and spring migration. Proceedings of the Royal Society B: Biological Sciences. 2012;279(1730):1008–1016. 10.1098/rspb.2011.1323 21900322 PMC3259926

[66] Kwan MP, Xiao N, Ding G. Assessing activity pattern similarity with multidimensional sequence alignment based on a multiobjective optimization evolutionary algorithm. Geographical Analysis. 2014;46:297–320. 10.1111/gean.12040 PMC4501399 26190858

[67] Eubank S, Guclu H, Kumar VA, Marathe MV, Srinivasan A, Toroczkai Z, et al Modelling disease outbreaks in realistic urban social networks. Nature. 2004;429(6988):180–184. 10.1038/nature02541 15141212

[68] Merler S, Ajelli M. The role of population heterogeneity and human mobility in the spread of pandemic influenza. Proceedings of the Royal Society B: Biological Sciences. 2010;277(1681):557–565. 10.1098/rspb.2009.1605 19864279 PMC2842687

[69] Dalziel BD, Pourbohloul B, Ellner SP. Human mobility patterns predict divergent epidemic dynamics among cities. Proceedings of the Royal Society B: Biological Sciences. 2013;280(1766):20130763 10.1098/rspb.2013.0763 23864593 PMC3730584

