# Supplementary material for: Spatial and Temporal Characteristics of Pastoral Mobility in the Far North Region, Cameroon: Data Analysis and Modeling
Source: PLoS One. 2015 Jul 7;10(7):e0131697. doi: 10.1371/journal.pone.0131697 (PMC4495066; doi:10.1371/journal.pone.0131697)
Supplement: S1 Text — (PDF) [file pone.0131697.s001.pdf]

Text S1. Classification of transhumant routes

We classify the transhumance routes shown in Figure 4 in the main text so that each group only contains routes that are geographically similar, meaning they have similar shapes and use the same areas for the rainy season locations and transhumance routes. We consider each route as a set of two dimensional coordinates of the points. These coordinates have a 2×2 covariance matrix that has two eigenvalues: the first eigenvalue indicates the amount of transformation of a circular shape along a long (major) axis and the second suggests the transformation along a short (minor) axis. This is similar to principal component analysis where we use an ellipse to fit the points. The length of the major axis of the ellipse is the first eigenvalue (e_1) and the length of the minor axis is the second eigenvalue (e_2). For our transhumance data, the major axis represents the main part a transhumance route that goes between rainy season lands and the floodplain. The minor axis represents how much pastoralists moved away from the main direction, which can be used to tell if the pastoralists used the paths east to Lake Maga.

To further illustrate the use of eigenvalues, let us consider two hypothetical routes shown in left plot of Figure A. Route 1 has a linear shape and its two eigenvalues are 4.00481028 and 0.02991195. Route 2 has a triangular shape and its two eigenvalues are 4.0150514 and 0.9492343. It is clear that the second eigenvalue of Route 2 is much greater than that of route1, which indicates a shape that is more stretched from its major axis.

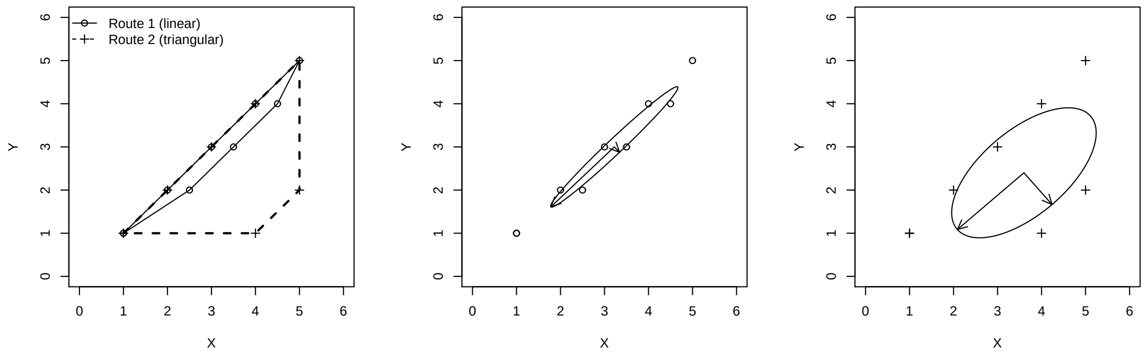

Figure A. Hypothetical routes and their eigenvalues. The left plot shows two routes. The ellipse that best fits Route 1 is shown in the center plot and that for Route 2 is in the right plot. Each ellipse is centered at the centroid of the points.

The ratio of the two eigenvalues can be used to indicate the shape of the annual path with a high ratio indicating a linear overall shape. We use the squared ratio of the two eigenvalues, $(\frac{e_1}{e_2})^2$, to ensure that routes represented by the red line in Figure 1 in the main text be distinguished from the other paths. Our test showed that the squared ratio of the lengths of the two axis is effective enough to separate first two groups of the pastoralists with a relatively linear shape and the third group. Figure A illustrates the use of eigenvalues on two hypothetical routes. For the two routes in Figure A, the squared ratios of the two eigenvalues for the two routes are significantly different: 17925.64 and 17.89103 for Route 1 and 2, respectively.

We conducted a hierarchical cluster analysis where the similarity of two routes is measured by the absolute difference between their squared ratios (note that the eigenvalues are standardized to the range between 0 and 1). Figure B shows the dendrogram of the analysis. At the height of 0.4, we have two clusters where the 7 pastoralists on the right all exhibit clear triangular shapes, and the other 64 pastoralists differ in their rainy season locations and whether they used areas near Lake Maga on their

transhumance routes, which can be further distinguished.

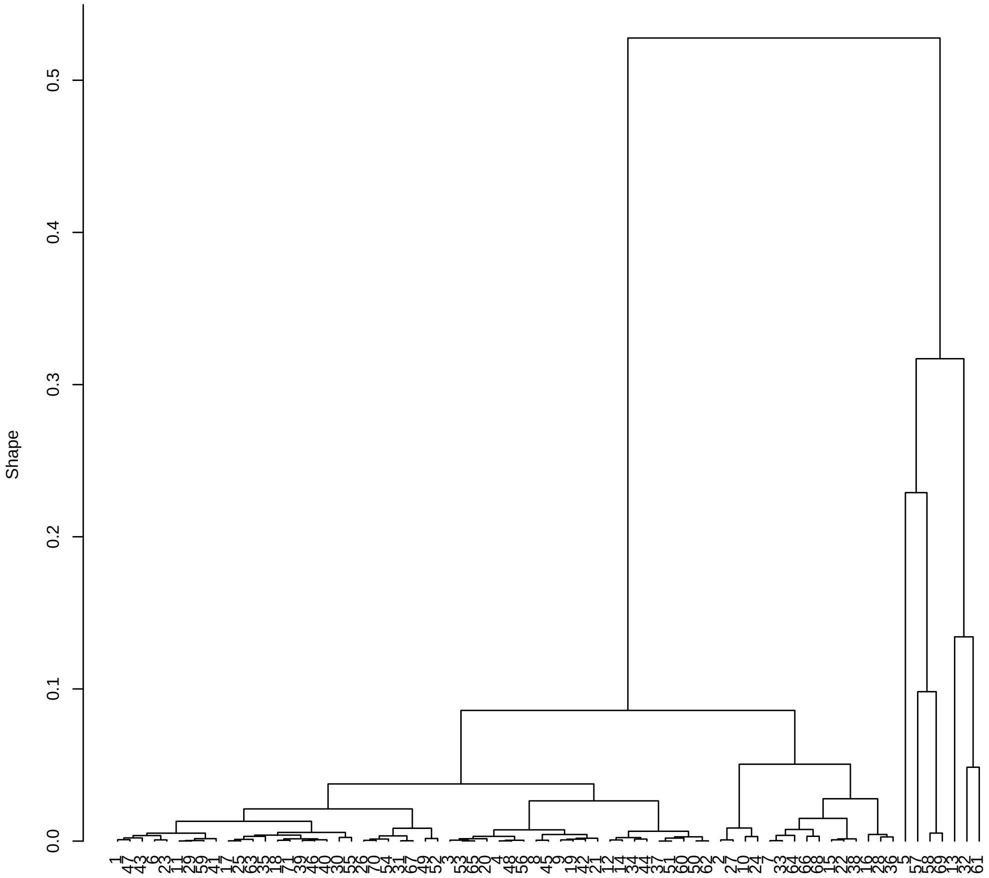

Figure B. Dendrogram showing the cluster analysis result of 71 pastoralists in 2007-2008 using the squared ratio of eigenvalues to measure the shape of each route.

We conducted a second round hierarchical cluster analysis using the rainy season location on August 16 and the centroid of all the locations visited by a pastoralist to measure the similarity between camps. Specifically, the similarity between camps i and j is measured as

$$\sqrt{(x_i^c - x_j^c)^2 + (y_i^c - y_j^c)^2 + (x_i^0 - x_j^0)^2 + (y_i^0 - y_j^0)^2}\,,$$

where x_i^c and y_i^c are the coordinates of the centroid and x_i^0 and y_i^0 are the coordinates on August 16 (note that the coordinates are standardized to range between 0 and 1). The use of the centroid is critical here as one group of pastoralists (e.g., green in Figure 1 in main text) have their centroid around Lake Maga and those in the other group (blue in Figure 1) do not. Here, we create 2 clusters using the height value

of 4 (Figure C). We call these two clusters Groups 1 and 2, and the cluster of routes with a triangular shape Group 3. The annual routes in these clusters are shown in Figure 5 in the main text.

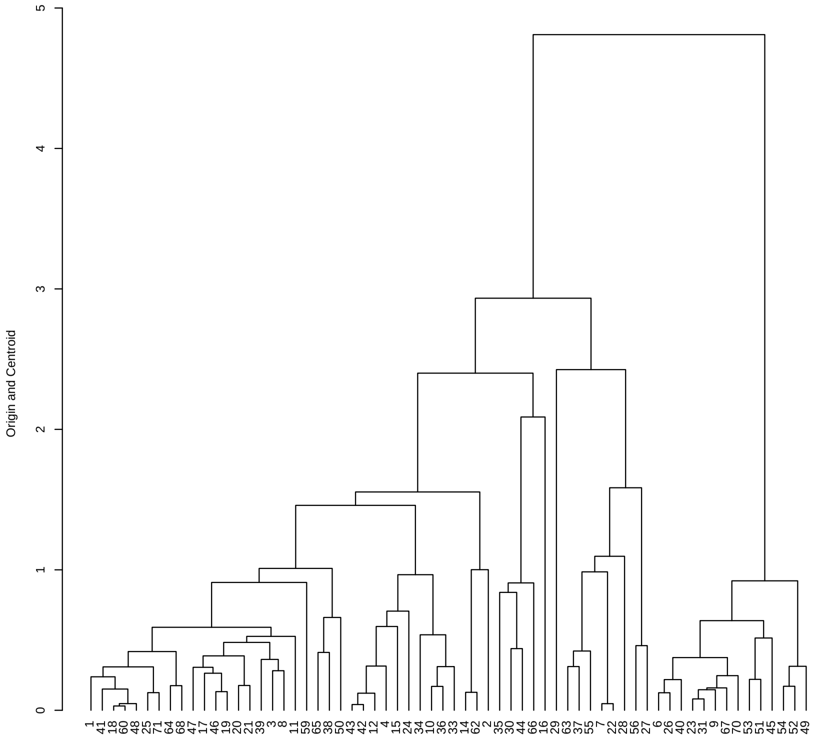

Figure C. Dendrogram showing the cluster analysis of 64 pastoralists in 2007-2008 using their rainy season locations and centroids.
