# Supplementary material for: Spatial and Temporal Characteristics of Pastoral Mobility in the Far North Region, Cameroon: Data Analysis and Modeling
Source: PLoS One. 2015 Jul 7;10(7):e0131697. doi: 10.1371/journal.pone.0131697 (PMC4495066; doi:10.1371/journal.pone.0131697)
Supplement: S2 Text — (PDF) [file pone.0131697.s002.pdf]

Text S2. Model comparison metrics

Three metrics are developed to test and evaluate the mobility models. These metrics are designed to measure the moving distance, closeness between pastoralists, and the spatial extent of the route, respectively.

First, we calculate the mean distance pastoralists make between two consecutive days as $D_t = \frac{1}{N}\sum_{i=1}^{N} d(x_{it}, x_{i(t-1)})$, where N is the number of pastoralists, x_{it} is the location of the i-th pastoralist at time t, and d is a function returning the Euclidean distance between two locations. The difference between the simulations and the data is calculated using the standardized root mean squared deviation of D_t between the simulated paths and that of the reported paths in the data for the entire year as $\sqrt{T\sum_{t=1}^{T}(D_t - D_t^*)^2} / \sum_t D_t^*$, where D_t^* is the mean moving distance at time t from the survey data, and T is the total time (354 days in this case).

Second, we measure the closeness of pastoralists at time t by computing the mean distance between the them at that time as $C_t = \frac{1}{2N(N-1)}\sum_{i=1}^{N}\sum_{j=i+1}^{N} d(x_{i,t}, x_{j,t})$. We then calculate the difference between the simulated routes and the data using the standardized root mean square deviation of C_t between the simulated paths and the reported in the data for the year as $\sqrt{T\sum_{t=1}^{T}(C_t - C_t^*)^2} / \sum_t C_t^*$, where C_t^* is the mean closeness at time t from the survey path data, and T is total time (365 days).

Lastly, we use the convex hull that encloses all the locations of the pastoralists in a group for the entire year as an indication of the overall spatial extent of the annual transhumance cycle. We measure the overlapped area ratio between the simulated routes and the data as $2a_o/(a + a')$, where a is the area of the convex hull of the locations in the data, a' is area of the convex hull from the simulated data, and a_o is the overlapped area between the two hulls. This measure is 1 if the two convex hulls are exactly the same, and is 0 if the two hulls do not intersect.
