# Supplementary material for: Spatial and Temporal Characteristics of Pastoral Mobility in the Far North Region, Cameroon: Data Analysis and Modeling
Source: PLoS One. 2015 Jul 7;10(7):e0131697. doi: 10.1371/journal.pone.0131697 (PMC4495066; doi:10.1371/journal.pone.0131697)
Supplement: S1 Fig — (PDF) [file pone.0131697.s003.pdf]

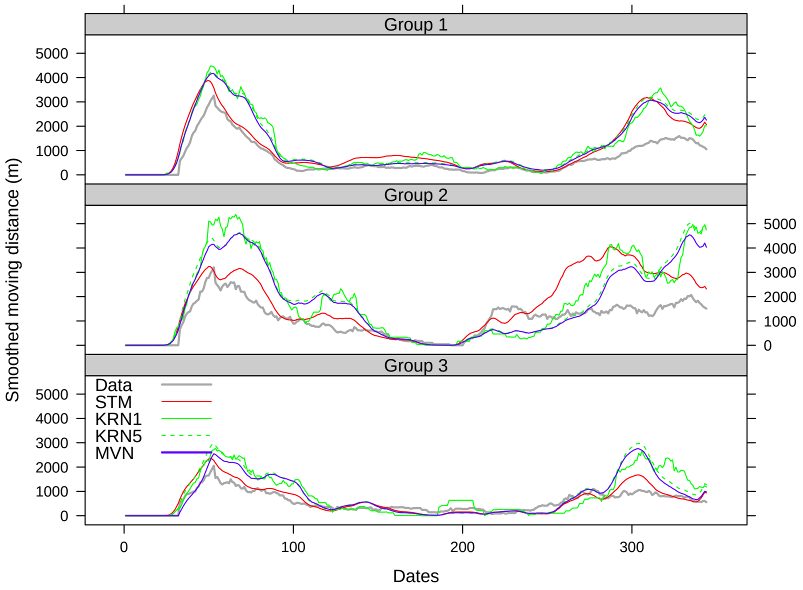

Figure S1. Smoothed daily mean moving distance obtained from the 2007-2008 data and 100 simulations using STM, KRN, and MVN.
