# Supplementary material for: Spatial and Temporal Characteristics of Pastoral Mobility in the Far North Region, Cameroon: Data Analysis and Modeling
Source: PLoS One. 2015 Jul 7;10(7):e0131697. doi: 10.1371/journal.pone.0131697 (PMC4495066; doi:10.1371/journal.pone.0131697)
Supplement: S1 Table — (PDF) [file pone.0131697.s004.pdf]

Table S1. Parameters estimated for the spatial and temporal models in the (STM). Note that the values for the mean locations in modes M3 and M4 are not included, because their values are computed using the estimates of the receding and following modes (see the Model Fitting section in the main text).

Group	Model	Transhumance Modes			
		M1	M2	M3	M4
1	Spatial	$\mu_1 = (461011\ 1153328)$<br>$\Sigma_1 = \begin{pmatrix} 201188160 & 113323428 \\ 113323428 & 267371989 \end{pmatrix}$	$\mu_2 = (504072\ 1224238)$<br>$\Sigma_2 = \begin{pmatrix} 25189074 & 6874746 \\ 6874746 & 280173504 \end{pmatrix}$	$\mu_{3,1,t},\ \mu_{3,2,t}$<br>$\Sigma_{3,1} = 550000,\ \Sigma_{3,2} = 550000$	
	Temporal	$\tau_1 = 10$<br>$\sigma_1^2 = 1010$	$\tau_2 = 236$<br>$\sigma_2^2 = 1030$	$\tau_{3,1} = 98$<br>$\sigma_{3,1}^2 = 40$<br>$\lambda_1 = 0.50$<br>$\tau_{3,2} = 323$<br>$\sigma_{3,2}^2 = 29$<br>$\lambda_2 = 0.50$	
2	Spatial	$\mu_1 = (432097\ 1205989)$<br>$\Sigma_1 = \begin{pmatrix} 95817006 & 30138356 \\ 30138356 & 24966798 \end{pmatrix}$	$\mu_2 = (496321\ 1224378)$<br>$\Sigma_2 = \begin{pmatrix} 74441678 & -76348025 \\ -76348025 & 194528969 \end{pmatrix}$	$\mu_{3,1,t},\ \mu_{3,2,t}$<br>$\Sigma_{3,1} = 550000,\ \Sigma_{3,2} = 550000$	
	Temporal	$\tau_1 = 19$<br>$\sigma_1^2 = 444$	$\tau_2 = 228$<br>$\sigma_2^2 = 796$	$\tau_{3,1} = 90$<br>$\sigma_{3,1}^2 = 29$<br>$\lambda_1 = 0.48$<br>$\tau_{3,2} = 304$<br>$\sigma_{3,2}^2 = 43$<br>$\lambda_2 = 0.52$	
3	Spatial	$\mu_1 = (443095\ 1135880)$<br>$\Sigma_1 = \begin{pmatrix} 80288178 & -34185421 \\ -34185421 & 575231228 \end{pmatrix}$	$\mu_{2,1} = (500598\ 1162592)$<br>$\Sigma_{2,1} = \begin{pmatrix} 5072936 & 26445587 \\ 26445587 & 286916864 \end{pmatrix}$<br>$w_1 = 0.31$<br>$\mu_{2,2} = (504344\ 1243159)$<br>$\Sigma_{2,2} = \begin{pmatrix} 72324481 & 131536200 \\ 131536200 & 1151711215 \end{pmatrix}$<br>$w_2 = 0.69$	$\mu_{3,1,t},\ \mu_{3,2,t}$<br>$\Sigma_{3,1} = 250000,\ \Sigma_{3,2} = 250000$	$\mu_{4,t}$<br>$\Sigma_4 = 250000$
	Temporal	$\tau_1 = 12$<br>$\sigma_1^2 = 1082$	$\tau_{2,1} = 210$<br>$\sigma_{2,1}^2 = 13$<br>$\lambda_1 = 0.51$<br>$\tau_{2,2} = 260$<br>$\sigma_{2,2}^2 = 18$<br>$\lambda_2 = 0.49$	$\tau_{3,1} = 96$<br>$\sigma_{3,1}^2 = 27$<br>$\lambda_1 = 0.44$<br>$\tau_{3,2} = 311$<br>$\sigma_{3,2}^2 = 39$<br>$\lambda_2 = 0.56$	$\tau_4 = 256$<br>$\sigma_4^2 = 401$
