# Supplementary material for: Spatial and Temporal Characteristics of Pastoral Mobility in the Far North Region, Cameroon: Data Analysis and Modeling
Source: PLoS One. 2015 Jul 7;10(7):e0131697. doi: 10.1371/journal.pone.0131697 (PMC4495066; doi:10.1371/journal.pone.0131697)
Supplement: S2 Table — (PDF) [file pone.0131697.s005.pdf]

Table S2. Results of t tests on STM versus the two reference models (5 km bandwidth for the KRN model). The numbers in the table are the p-values from each of the tests.

Year	Group	Closeness		Moving distance		Overlapped area ratio	
		v. KRN5	v. MVN	v. KRN5	v. MVN	v. KRN5	v. MVN
2007-2008	1	0.000	0.000	0.000	0.001	*0.067*	0.000
	2	0.000	0.000	0.000	0.014	0.000	0.000
	3	*0.934*	*1.000*	0.000	0.000	*0.999*	0.018
2008-2009	1	0.000	0.000	0.000	0.000	0.000	*0.506*
	2	0.000	0.000	0.000	0.000	0.000	*0.904*
	3	0.000	0.000	0.000	0.000	*1.000*	*1.000*
2009-2010	1	0.000	0.000	0.000	0.000	0.035	*1.000*
	2	0.000	0.000	0.000	0.003	0.000	0.000
	3	0.000	0.000	0.000	0.000	*1.000*	*1.000*
2010-2011	1	*1.000*	*1.000*	0.000	0.000	0.000	0.000
	2	0.000	0.000	0.000	0.000	0.000	0.000
	3	0.000	0.000	0.000	0.000	*1.000*	*1.000*
